# The Hypoxia-Related Gene COL5A1 Is a Prognostic and Immunological Biomarker for Multiple Human Tumors

**DOI:** 10.1155/2022/6419695

**Published:** 2022-01-17

**Authors:** Hua Zhu, Xinyao Hu, Shi Feng, Zhihong Jian, Ximing Xu, Lijuan Gu, Xiaoxing Xiong

**Affiliations:** ^1^Department of Neurosurgery, The Affiliated Huzhou Hospital, Zhejiang University School of Medicine (Huzhou Central Hospital), Huzhou, China; ^2^Department of Neurosurgery, Renmin Hospital of Wuhan University, Wuhan, China; ^3^Cancer Center, Renmin Hospital of Wuhan University, Wuhan, China; ^4^Central Laboratory, Renmin Hospital of Wuhan University, Wuhan, China; ^5^Department of Anesthesiology, Renmin Hospital of Wuhan University, Wuhan, China

## Abstract

**Background:**

Collagen type V alpha 1 chain (COL5A1) is a hypoxia-related gene (a collagen family protein) and participates in the formation of the extracellular matrix. Although some evidence supports a significant role for COL5A1 in the progression of several cancers, a pan-cancer analysis of COL5A1 is not currently available. Herein, we aimed to assess the prognostic value of COL5A1 in 33 human cancers and to investigate its underlying immunological function.

**Methods:**

Through multiple bioinformatics methods, we analyzed the data from Oncomine, TCGA, CCLE, HPA, DNMIVD, and cBioPortal database to explore the potential underlying carcinogenic effect of COL5A1, including the relevance of COL5A1 to the outcome, DNA methylation, tumor microenvironment, immune cells infiltration, and drug sensitivity in 33 human cancers. The effects of COL5A1 on glioma cell proliferation, migration, and invasion were verified in cellular experiments.

**Results:**

Our findings indicated that COL5A1 was expressed at high levels in 13 cancers and was negatively related to the prognosis of 11 cancers. Additionally, COL5A1 was coexpressed with genes encoding the major histocompatibility complex, immune activators, immune suppressors, chemokines, chemokine receptors, mismatch repair genes, and immune checkpoints. We also identified different roles for COL5A1 in the immunocyte infiltration in different cancers. The correlation between COL5A1 and drug sensitivity was found in several cancers. COL5A1 potentially influenced the tumor progression through immune-related pathways, negative regulation of immune system processes, chemokine signaling pathways, JAK-STAT pathways, T cell receptor pathways, lymphocyte migration, and antigen processing and presentation, among other processes.

**Conclusions:**

Based on our study, COL5A1 may be employed as a prognostic marker in different malignancies because of its impact on tumorigenesis and immune cell infiltration and have implications for cancer immune checkpoint inhibitors and chemotherapy.

## 1. Introduction

Malignant tumor is a leading cause of death and a main cause of poor quality of life of patients in many countries worldwide, but no absolute cure is currently available for malignant tumors [1], such as central nervous tumors with neurorestorative treatment [2, 3]. Immunotherapy for tumors has recently emerged as a new approach to oncology treatment, specifically immune checkpoint blockade (ICB) therapy [4]. The emergence and refinement of gene expression databases have enabled explorations of new immunotherapeutic targets through pan-cancer analysis of the expression of particular gene and assessments of its relevance to patients' prognoses and potential mechanisms [5, 6].

Collagen type V alpha 1 chain (COL5A1), a collagen family protein (the most abundant matrix protein polymer in vertebrates), participates in the formation of extracellular matrix [7]. Additionally, COL5A1 was also reported to be a hypoxia-related gene [8]. Hypoxia is known to trigger the production of reactive oxygen species [9], which play an important role in cancer biology. Collagen deposition is often regarded as a pathological characteristic in tumor microenvironment [10]. In addition, chemotherapy resistance is associated with increased tissue stiffness mediated by specific collagen cross-linking. Collagen V is one of the components of fibril-forming collagen and has a critical role in extracellular matrix organization by forming copolymers with collagen I or II to regulate the length and abundance of heterotypic collagenous protofibrils [11]. *α*1(V), *α*2(V), and *α*3(V) polypeptide chains, encoded by COL5A1, COL5A2, and COL5A3, respectively, comprise the protein of collagen V [12]. As previously reported, COL5A1 is associated with head and neck squamous cell carcinoma (HNSC), oral squamous cell carcinoma [13], breast cancer [14], and gastric cancer [15]. Furthermore, COL5A1 has recently been reported to be a key gene correlated with macrophage infiltration and M2 polarization and is related to the proportion of infiltrating immunocyte in ovarian cancer (OV), indicating that COL5A1 may be an immunotherapeutic target in OV [16]. However, the role of COL5A1 in other human cancers is still unidentified. The prognostic predictive value of COL5A1 across cancers has not been adequately studied. Therefore, more work is urgently needed to investigate the role of COL5A1 in human tumors.

Tumor-infiltrating immune cells (TIIC) are important components of the tumor microenvironment (TME) and monitor tumor cells during their life cycle, and cancer only develops when immune cells cannot destroy precancerous cells [17]. The infiltration levels of TIIC in TME also impact on the prognoses of cancers. For example, high levels of B, CD4+, and dendritic cells infiltration are associated with a better outcome in thymomas, which may be partially regulated by ASF1B [6]. Lower grade glioma (LGG) patients with high TUBA1C expression may have a better response to ICB [18]. More and more versatile immunotherapeutic targets for human tumors need to be discovered. Herein, we mainly used numerous silico analyses to discover the role of COL5A1 in the prognosis, TIIC infiltration, and drug sensitivity in 33 human cancers.

## 2. Methods

### 2.1. Data Collection and Different Expression Analysis

Oncomine (https://www.oncomine.org/), an online cancer microarray database [19], was employed to analyze the COL5A1 expression in 33 human tumors as we used to do [20]. We downloaded mRNA expression data and clinical information from the UCSC Xena website (https://xena.ucsc.edu/). Next, we extracted and integrated COL5A1 expression data in TCGA (https://tcga.xenahubs.net) through the Perl software to perform pan-cancer analysis. The “Wilcoxon test” was employed to evaluate the differences in COL5A1 mRNA expression levels in 33 cancer types. Then, mRNA sequencing data from different cancer cell lines were assessed using CCLE (https://portals.broadinstitute.org/ccle). The R package “ggpubr” was employed for the box diagram. Mutation in COL5A1 in 33 cancers was investigated through cBioPortal (https://www.cbioportal.org).

### 2.2. Immunohistochemical (IHC) Staining

Images of immunohistochemical staining for the COL5A1 protein were used for expression analyses, and the differences in the COL5A1 protein level were assessed in normal and eight tumor tissues, including bladder urothelial carcinoma (BLCA), colon adenocarcinoma (COAD), glioblastoma multiforme (GBM), liver hepatocellular carcinoma (LIHC), ovarian cancer (OV), prostate adenocarcinoma (PRAD), stomach adenocarcinoma (STAD), and testicular germ cell tumors (TGCTs) from the HPA (http://www.proteinatlas.org/). Three normal tissue and five tumor tissue samples were randomly chosen for quantitative analysis.

### 2.3. Western Blot Analysis

U251 cells, tumor, and paracancerous tissues were collected (representative clinicopathological data are presented in Figure S1), homogenized, and then lysed on ice in cold RIPA buffer. Next, the samples were separated on gels and then transferred onto polyvinylidene difluoride membranes. Membranes were blocked by blocking buffer for 1 hour and then incubated with anti-COL5A1 (Sigma-Aldrich; Merck KGaA) and anti-*β*-tubulin (Cell Signaling Technology, Boston, USA) primary antibodies at 4°C overnight. Membranes were then incubated with secondary antibody (diluted 1 : 10000; Li-Cor Bioscience, USA) for 1 hour. Membranes were assessed with the Odyssey software (LI-COR, Lincoln, NE, USA).

### 2.4. Identification of the Correlation between COL5A1 Expression and the Clinicopathological Characteristics or Survival of Patients with Various Cancers

Survival information for each sample in TCGA was used to elucidate the relationship between COL5A1 expression and prognosis of patients with different cancers. Overall survival (OS), disease-specific survival (DSS), disease-free interval (DFI), and progression-free interval (PFI) were analyzed. The Kaplan–Meier (KM) survival curves and log-rank test were employed to analyze the survival of patients (*p* < 0.05) through R packages “survminer” and “survival.” Subsequently, “survival” and “forestplot” R packages were applied for the Cox analysis to verify the correlation of COL5A1 expression with survival. High or low expressions are defined by median. R packages “ggpubr” and “limma” were employed for the clinicopathological correlation analysis. The DNMIVD dataset (http://www.unimd.org/dnmivd/) was applied to assess the DNA methylation levels of COL5A1 and the association between COL5A1 methylation and prognosis.

### 2.5. Correlation Analysis of COL5A1 with Tumor Mutation Burden (TMB) or Microsatellite Instability (MSI)

The correlation of COL5A1 with TMB or MSI was analyzed as previously described [6, 20]. We also applied cBioPortal to investigate the correlation between COL5A1 mutations and prognosis.

### 2.6. Exploration of Association between COL5A1 and TME or Infiltration of TIIC

We employed the ESTIMATE algorithm and the R packages “estimate” and “limma” to evaluate immune and stromal scores [21] as we used to [6, 20, 22]. For more reliable immune score evaluation, we then employed the “immuneeconv” R package that integrated the six algorithms, including TIMER, xCell, MCP-counter, CIBERSORT, EPIC, and quanTIseq. We next analyzed the correlation of COL5A1 expression with the TME or TIIC infiltration by R packages “ggplot2,” “ggpubr,” and “ggExtra.”

### 2.7. Pathway Enrichment Analysis and Coexpression of COL5A1 with Immune-Related, Mismatch Repair (MMR) Genes, and Immune Checkpoint-Related Genes in Tumors

Gene Ontology (GO) and Kyoto Encyclopedia of Genes analyses and Genomes (KEGG) and the coexpression of COL5A1 with immune checkpoint genes and immune-related genes were conducted as we described previously [6, 20].

### 2.8. Predicting the Correlation of COL5A1 with Drug Sensitivity

We predicted the chemotherapeutic response for samples of cancers which had a correlation of COL5A1 with OS based on the Genomics of Drug Sensitivity in Cancer (GDSC) (https://www.cancerrxgene.org/). The prediction was implemented by the R package “pRRophetic.” All parameters were set by the default values with removal of the batch effect of “combat” and tissue type of “allSoldTumours,” and we generalized duplicate gene expression to mean value [23].

### 2.9. qRT-PCR, Wound Healing, and Transwell Migration to Confirm the Role of COL5A1 in U251 Cells

U251 cell line was obtained from Genochem (Shanghai, China). Cells were cultured in DMEM with 10% FBS and 100 U/mL penicillin-streptomycin in an incubator at 37°C with 5% CO2. The sequence of siRNA to knock down COL5A1 was 5′-AAGGAGAGGGUGAGACCUAUUA-3′, and the sequence for control siRNA was 5′-CAGAGGGAGUGGGAGCCAAUAAUUA-3′ [24]. Lipofectamine 3000 was employed for cell transfection with siRNA-COL5A1 or siRNA-control. 48 hours after the transfection process, transfected cells were collected, and then the transfection efficiency was verified by western blotting and qRT-PCR assays. Then, the cells were plated in six-well plates until fused to 80%. The cells were scratched using a pipette tip and washed, then incubated at 37°C and 5% CO2. The viability of U251 cells was assessed by CCK-8 assay. After 24 h, the picture of the wound was taken under a microscope.

qRT-PCR was carried out as a previous study [25]. The primer sequences were as follows: COL5A1, F: GCCCGGATGTCGCTTACAG, R: AAATGCAGACGCAGGGTACAG; GAPDH, F: GCACCGTCAAGGCTGAGAAC, R: TGGTGAAGACGCCAGTGGA. The mRNA expression was quantified by normalizing it with the internal reference GAPDH expression.

Cell invasion assay was performed as we previously performed [26]. The cells were photographed at 200X magnification, and the number cells crossing membrane was counted in five random fields by ImageJ (version 1.61, NIH, Bethesda, MD, USA).

### 2.10. Statistical Analysis

All gene expression data were normalized by log2 transformation. Experimental data were presented as mean ± SD, and Student's *t*-test was employed for two-group comparison. KM analysis, Cox proportional hazards model, and log-rank test were performed for all survival analyses. Correlation between two variables was evaluated by Pearson's or Spearman's test. Statistical analyses were carried out using GraphPad Prism 8.0 (GraphPad Software Inc.) and R software (version 4.0.2). *p* < 0.05 was defined as a significant difference.

## 3. Results

### 3.1. Differential Expression of COL5A1 in Normal and Tumor Tissues

**Figure 1:**
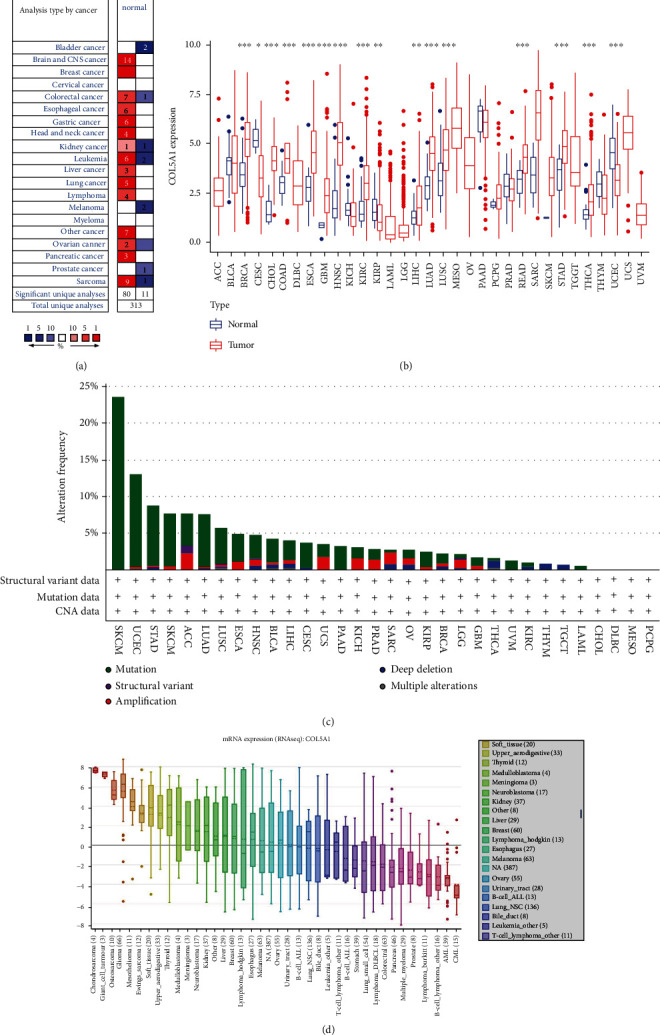
Differential COL5A1 expression in normal and tumor tissues. (a) COL5A1 mRNA expression was elevated in most tumors than normal tissues. (b) Different levels of COL5A1 expression in various human tumors and normal tissues. (c) The alteration frequency of COL5A1 in different cancers. (d) Expression of the COL5A1 mRNA in various cell lines. ^∗^*p* < 0.05, ^∗∗^*p* < 0.01, and ^∗∗∗^*p* < 0.001.

Firstly, Oncomine was used to assess the COL5A1 expression in normal and tumor tissues. We found that COL5A1 was highly expressed in most human cancers, including the brain and central nervous system (CNS), breast, colorectal, esophageal, gastric, head and neck, kidney, leukemia, liver, lung, lymphoma, ovarian, pancreatic, sarcoma, and other cancers. It was interesting to note that lower expression of COL5A1 was also detected in bladder, colorectal, kidney, leukemia, melanoma, ovarian, sarcoma, and prostate cancer datasets (Figure 1(a)). Different data collection methods may be partially responsible for these contradictory results.

We analyzed the RNA sequencing data from TCGA through using the R software to further evaluate the expression of COL5A1 in different cancers. The results illustrated that COL5A1 was upregulated in 13 cancers, including breast invasive carcinoma (BRCA), cholangiocarcinoma (CHOL), COAD, esophageal carcinoma (ESCA), GBM, HNSC, kidney renal clear cell carcinoma (KIRC), LIHC, lung squamous cell carcinoma (LUSC), lung adenocarcinoma (LUAD), rectal adenocarcinoma (READ), STAD, and thyroid carcinoma (THCA). Meanwhile, lower COL5A1 expression was detected in 3 cancers compared to normal tissues, including cervical squamous cell carcinoma (CESC), uterine corpus endometrial carcinoma (UCEC), and kidney renal papillary cell carcinoma (KIRP). No significant difference was observed in some cancers with a few normal samples (e.g., only one normal tissue sample from patients with skin cutaneous melanoma (SKCM)); this may be because of the small sample size (Figure 1(b)). Then, we evaluated the changes in COL5A1 based on the cBioPortal database. COL5A1 expression was altered in 531 of 10,953 patients (~5%) included in TCGA. The highest alteration ratio was related to mutation, followed by amplifications and deep deletions. SKCM presented the highest alteration frequency among all cancers. Meanwhile, we assessed the mRNA sequence of COL5A1 in 33 cancers in the CCLE database. The five cancer cell lines with the highest mRNA expression of COL5A1 were chondrosarcoma, giant cell tumor, osteosarcoma, glioma, and mesothelioma (Figure 1(d)).

**Figure 2:**
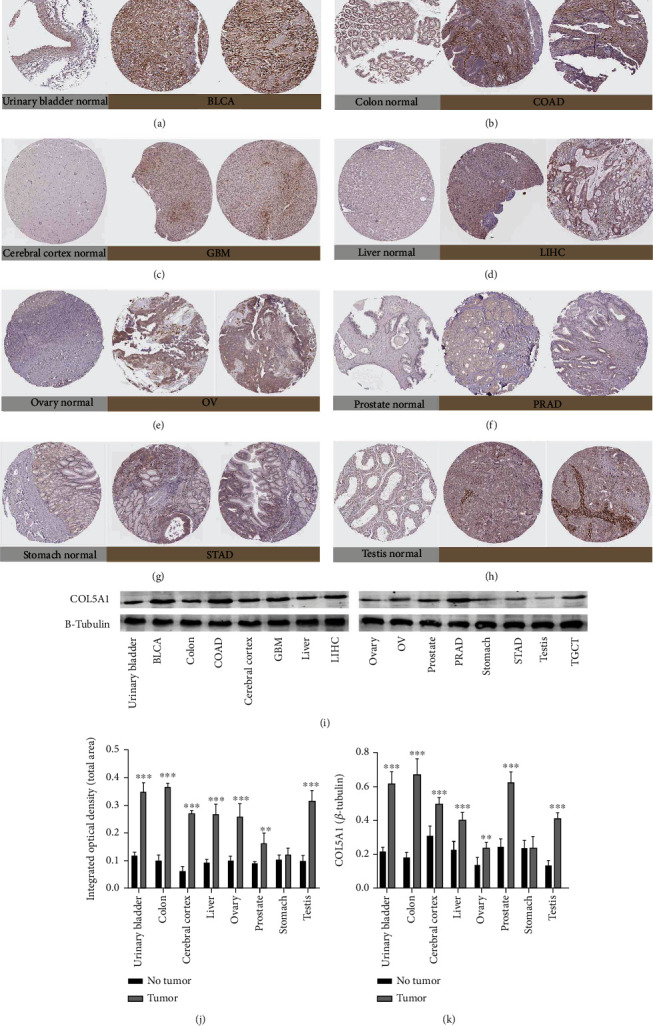
Representative photographs of immunohistochemical staining and western blots of different normal tissues (left panels) and tumor tissues (right panels). The level of the COL5A1 protein was increased in bladder urothelial carcinoma (BLCA), colon adenocarcinoma (COAD), glioblastoma multiforme (GBM), liver hepatocellular carcinoma (LIHC), ovarian cancer (OV), prostate adenocarcinoma (PRAD), and testicular germ cell tumors (TGCTs). (a) Urinary bladder. (b) Colon. (c) Cerebral cortex. (d) Liver. (e) Ovary. (f) Prostate. (g) Stomach. (i) Testis. (j) Quantitative analysis of immunohistochemical staining from the HPA database. *n* = 3 samples per normal group, *n* = 5 samples per tumor group. (i, k) Western blot results showing the expression of COL5A1, *n* = 5, ^∗∗^*p* < 0.01 and ^∗∗∗^*p* < 0.001.

Subsequently, the IHC photographs in HPA dataset were analyzed to investigate the expression of the COL5A1 protein. The results from HPA and TCGA were consistent with each other. COL5A1 IHC staining was weak in the normal ovary, cerebral cortex, liver, prostate, and testis, while GBM, LIHC, PRAD, and TGCTs showed low COL5A1 IHC staining and OV exhibited moderate COL5A1 IHC staining. COL5A1 IHC staining was weak in the normal urinary bladder, colon, and stomach, while BLCA and COAD displayed strong COL5A1 IHC staining and STAD presented weak COL5A1 IHC staining (Figures 2(a)–2(h) and 2(j)). We further collected tissues for western blots, and the results verified the IHC staining from the HPA database (Figures 2(i) and 2(k)).

### 3.2. Prognostic Value of COL5A1 in Various Cancers

**Figure 3:**
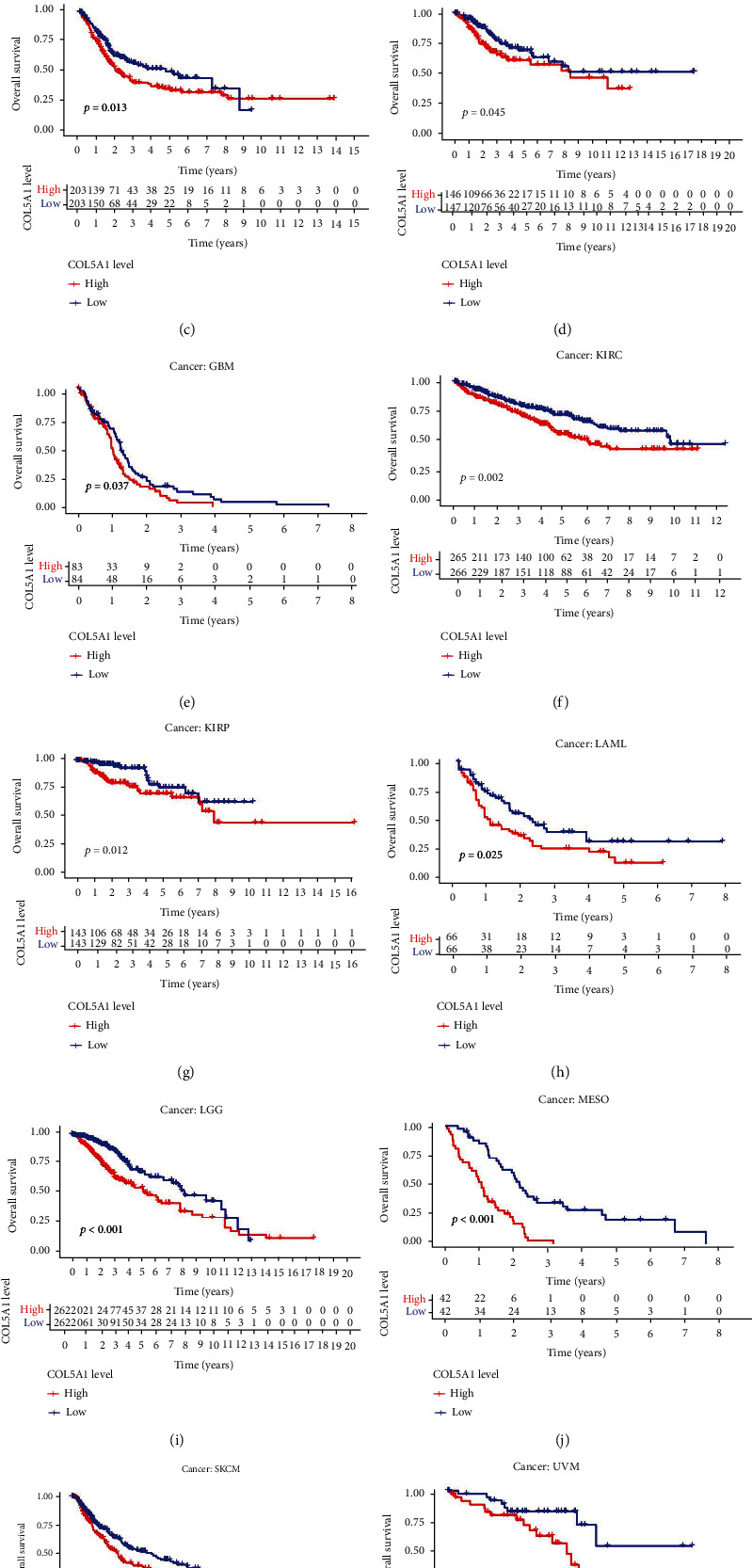
Correlations of COL5A1 with OS. (a) Forest plots and KM analyses (b–l) of the correlations of COL5A1 with OS in various human cancers.

Survival analyses, including OS, DSS, DFI, and PFI, were carried out for patients with 33 cancers to investigate the correlation between COL5A1 expression and prognosis. The Cox analysis illustrated that COL5A1 expression was closely correlated with the OS of patients with adrenocortical carcinoma (ACC), GBM, kidney chromophobe (KICH), KIRC, KIRP, LGG, LUAD, mesothelioma (MESO, pancreatic adenocarcinoma (PAAD), SKCM, STAD, THCA, and uveal melanoma (UVM) (Figure 3(a)). Furthermore, COL5A1 was a high-risk gene in these cancers, particularly UVM (hazard ratio = 2.426). In addition, KM plotter survival curves demonstrated that among the patients with ACC (Figure 3(b), *p* = 0.01), BLCA (Figure 3(c), *p* = 0.013), CESC (Figure 3(d), *p* = 0.045), GBM (Figure 3(e), *p* = 0.037), KIRC (Figure 3(f), *p* = 0.002), KIRP (Figure 3(g), *p* = 0.012), acute myeloid leukemia (LAML) (Figure 3(h), *p* = 0.025), LGG (Figure 3(i), *p* < 0.001), MESO (Figure 3(j), *p* < 0.001), SKCM (Figure 3(k), *p* = 0.005), and UVM (Figure 3(l), *p* = 0.023), those with high COL5A1 expression experienced a shorter survival.

**Figure 4:**
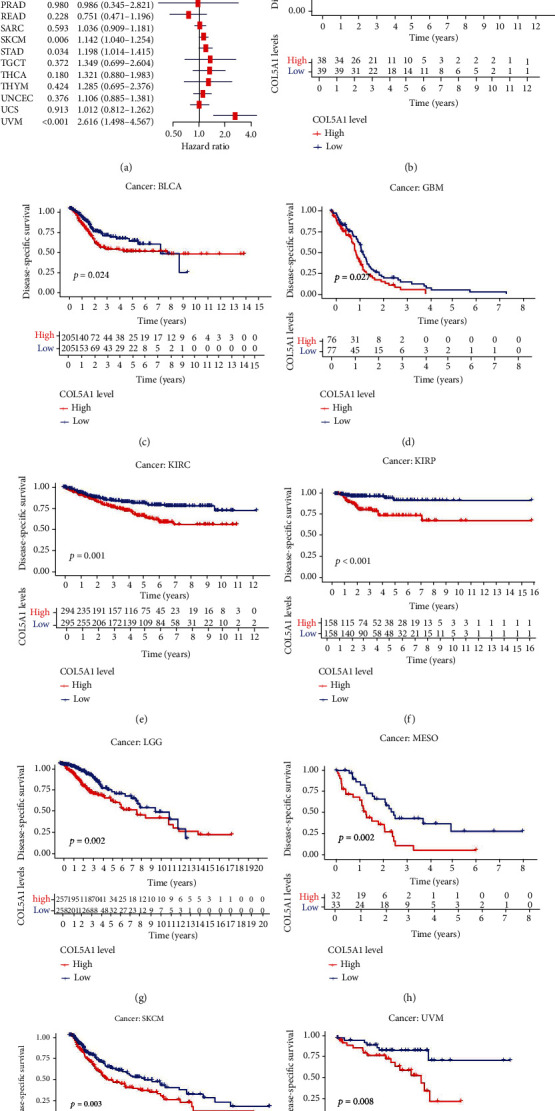
Correlations between COL5A1 and DSS. (a) Forest plots and KM analyses (b–j) of the relationships between COL5A1 and DSS.

**Figure 5:**
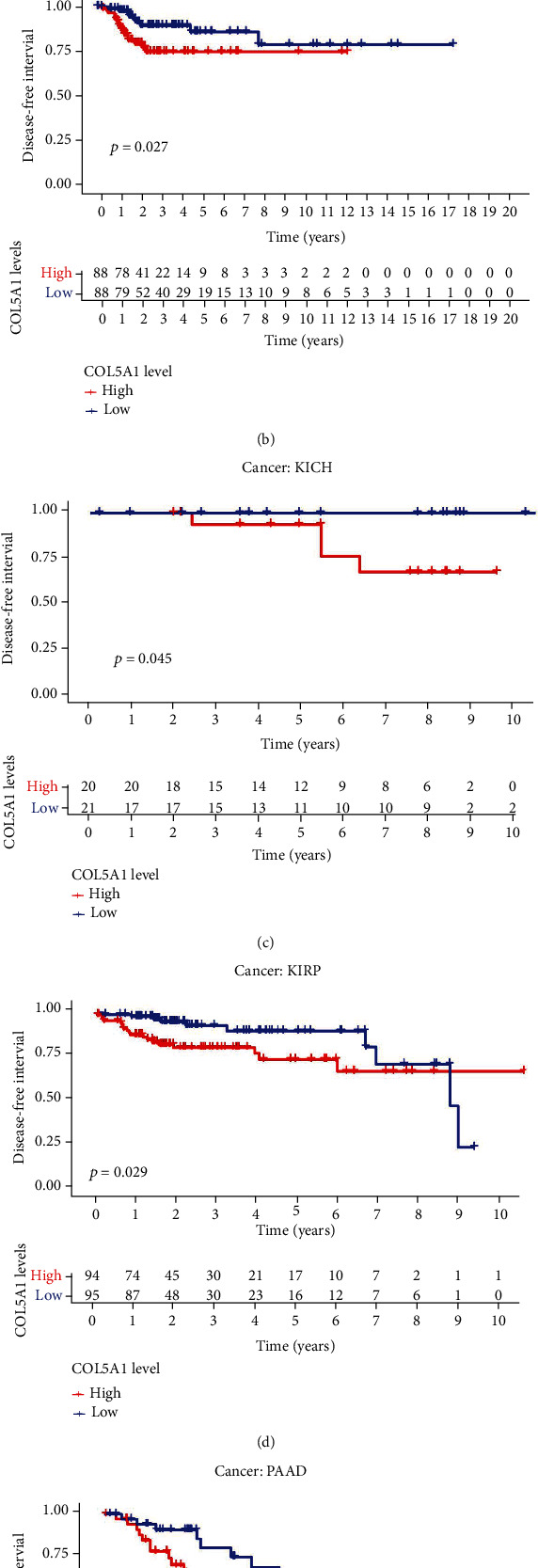
Correlations between COL5A1 and DFI. (a) Forest plots and KM analyses (b–e) of the relationships between COL5A1 and DFI.

**Figure 6:**
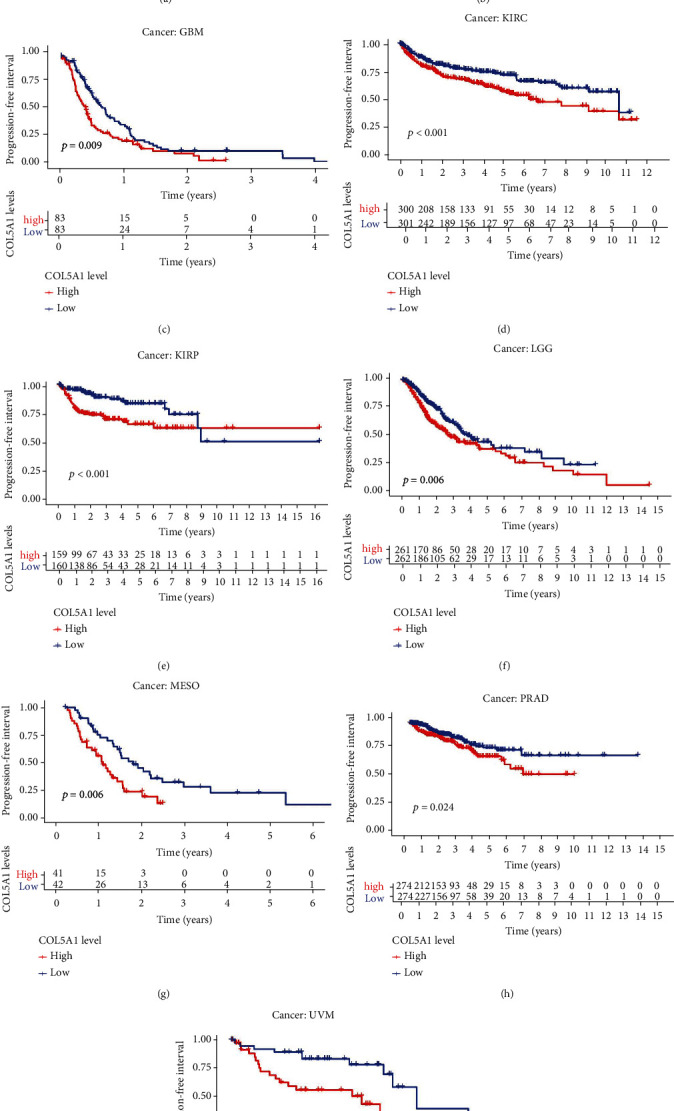
Correlation between COL5A1 and PFI. (a) Forest plots and KM analyses (b–i) of the relationships of COL5A1 with PFI.

Moreover, in the patients with ACC, BLCA, GBM, KICH, KIRC, KIRP, LGG, MESO, PAAD, SKCM, STAD, and UVM, DSS survival analyses verified a correlation between high COL5A1 expression and poor prognosis (Figure 4(a)). KM analyses also revealed a correlation between high COL5A1 expression levels and adverse outcome of patients with ACC (Figure 4(b), *p* = 0.029), BLCA (Figure 4(c), *p* = 0.024), GBM (Figure 4(d), *p* = 0.027), KIRC (Figure 4(e), *p* < 0.001), KIRP (Figure 4(f), *p* < 0.001), LGG (Figure 4(g), *p* = 0.002), MESO (Figure 4(h), *p* = 0.002), SKCM (Figure 4(i), *p* = 0.003), and UVM (Figure 4(b), *p* = 0.008). Correlations of high COL5A1 expression with a poor DFI were revealed in patients with ACC, CESC, KIRP, LUA, and PAAD (Figure 5(a)). Additionally, the KM analysis revealed significant correlations between COL5A1 with DFI in CESC (Figure 5(b), *p* = 0.027), KICH (Figure 5(c), *p* = 0.045), KIRP (Figure 5(d), *p* = 0.029), and PAAD (Figure 5(e), *p* = 0.015). In addition, forest plots demonstrated the relationship between high COL5A1 expression and a poor PFI in patients with ACC, BLCA, CESC, GBM, KICH, KIRC, KIRP, LGG, LUAD, MESO, PAAD, PRAD, and UVM (Figure 6). KM analyses illustrated that patients with ACC (Figure 6(b), *p* = 0.034), GBM (Figure 6(c), *p* = 0.009), KIRC (Figure 6(d), *p* < 0.001), KIRP (Figure 6(e), *p* < 0.001), LGG (Figure 6(f), *p* = 0.006), MESO (Figure 6(g), *p* = 0.006), PRAD (Figure 6(h), *p* = 0.024), and UVM (Figure 6(i), *p* = 0.002) who exhibited low expression of COL5A1 had longer survival time.

### 3.3. Correlations between COL5A1 Expression and Clinicopathology in Human Tumors

**Figure 7:**
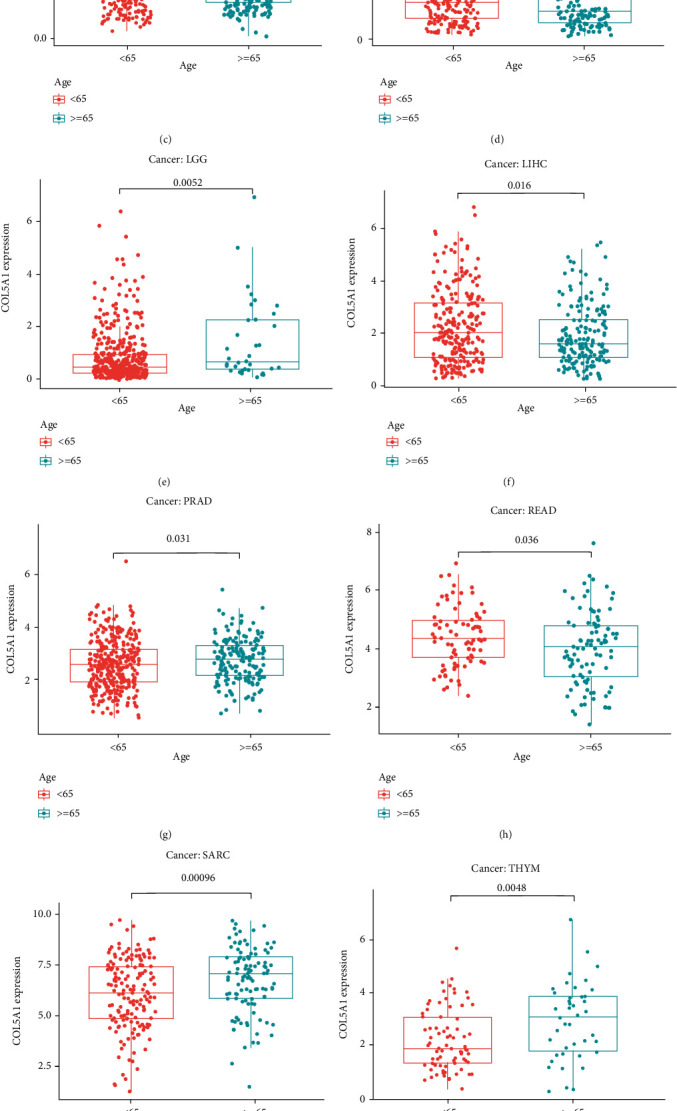
Relationships between COL5A1 and age in patients with (a) BLCA, (b) CESC, (c) KIRC, (d) KIRP, (e) LGG, (f) LIHC, (g) PRAD, (h) READ, (i) SARC, (j) THYM, and (k) UCEC.

Subsequently, we investigated the differences in COL5A1 expression levels in patients with different tumors stratified based on age and revealed that patients aged ≥ 65 years with CESC (Figure 7(b), *p* = 0.046), KIRC (Figure 7(c), *p* = 0.017), KIRP (Figure 7(d), *p* = 0.049), LIHC (Figure 7(f), *p* = 0.016), READ (Figure 7(h), *p* = 0.036), and UCEC (Figure 7(k), *p* < 0.001) had lower expression of COL5A1, while patients aged ≥ 65 with BLCA (Figure 7(a), *p* = 0.028), LGG (Figure 7(e), *p* = 0.0052), PRAD (Figure 7(g), *p* = 0.031), SARC (Figure 7(i), *p* < 0.001), and THYM (Figure 7(j), *p* = 0.048) displayed higher expression of COL5A1 than patients aged < 65 years. However, no obvious correlation was observed between age and COL5A1 expression in other cancers (Figure S2).

**Figure 8:**
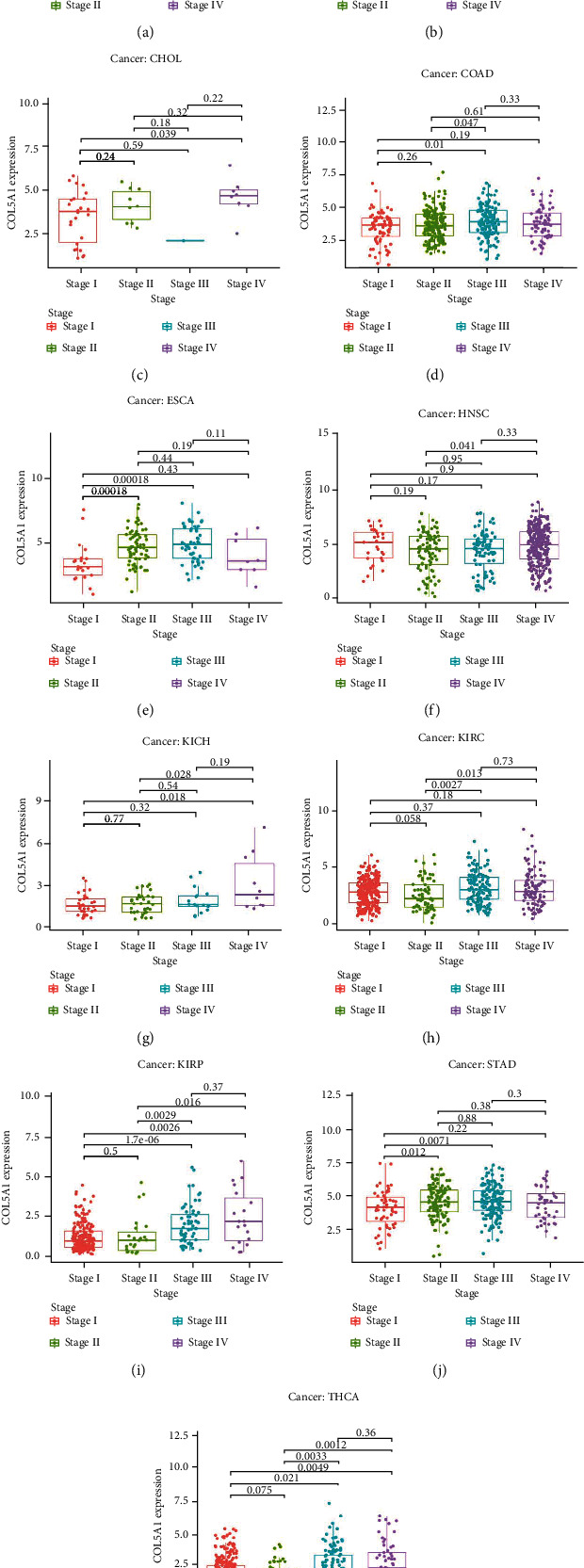
Associations between COL5A1 expression and the tumor stage in (a) ACC, (b) BLCA, (c) CHOL, (d) COAD, (e) ESCA, (f) HNSC, (g) KICH, (h) KIRC, (i) KIRP, and (j) STAD and THCA.

We analyzed the correlation between COL5A1 and tumor stage and found that COL5A1 expression was significantly correlated to the tumor stage in 13 human tumors, including ACC, BLCA, CHOL, COAD, ESCA, HNSC, THCA, KICH, KIRC, KIRP, STAD, PAAD, and UVM (Figure 8; Figure S3). Notably, in patients with CHOL (Figure 8(c), *p* = 0.039), KICH (Figure 8(g), *p* = 0.018), KIRP (Figure 8(i), *p* = 0.0026), and THCA (Figure 8(k), *p* = 0.0049), COL5A1 expression was significantly increased in stage IV tumors than stage I tumors. In addition, COL5A1 was also expressed at higher levels in stage III tumors than in stage I tumors in COAD (Figure 8(d), *p* = 0.01), ESCA (Figure 8(e), *p* < 0.001), KIRC (Figure 8(h), *p* = 0.036), KIRP (Figure 8(i), *p* < 0.001), STAD (Figure 8(j), *p* = 0.0071), and THCA (Figure 8(k), *p* = 0.021). COL5A1 was overexpressed in stage IV tumors than in stage II tumors in patients with ACC (Figure 8(a), *p* = 0.0015), BLCA (Figure 8(b), *p* < 0.001), HNSC (Figure 8(f), *p* = 0.041), KICH (Figure 8(g), *p* = 0.028), KIRC (Figure 8(h), *p* = 0.013), KIRP (Figure 8(i), *p* = 0.016), and THCA (Figure 8(k), *p* = 0.0012). Therefore, we hypothesized that high COL5A1 expression may lead to shorter survival time in these patients with advanced cancer. Although the differences were remarkable between stages I and IV, stages I and III, and stages II and IV, the differences between stages in other cancers were comparatively small (Figure 8, Figure S3), and a statistically significant difference was not observed in other human tumors (Figure S3).

### 3.4. Associations of COL5A1 Expression with TMB, MSI, Mismatch Repair Genes, and the Mutation-Related Prognosis in Various Human Tumors

**Figure 9:**
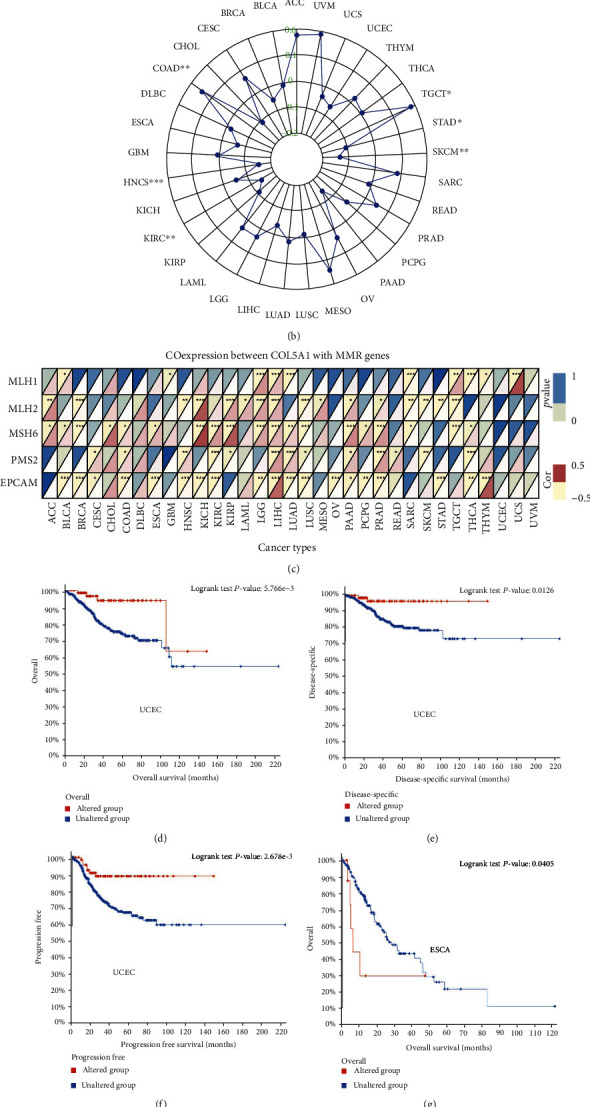
Correlations of COL5A1 expression with the TMB, MSI, MMR genes, and mutation-related prognosis in various human cancers. (a) Radar plot showing the relationship between COL5A1 expression and TMB in human cancers. (b) Radar plot showing the relationship between COL5A1 expression and MSI across human cancers. (c) Coexpression of COL5A1 with MMR genes. Correlations of COL5A1 gene mutations with (d) OS, (e) DSS, and (f) PFI of patients with UCEC. Correlations of COL5A1 gene mutations with (g) OS and (h) PFI of patients with ESCA.

We investigated the associations between COL5A1 expression and the TMB and MSI, both of which are involved in the sensitivity to ICB. Therefore, an investigation of the relationships between the TMB and COL5A1 across cancers is necessary. COL5A1 expression was related to the TMB in 13 cancer types. In particular, COL5A1 expression positively linked to the TMB in 4 tumors, including ACC, LAML, LGG, and THYM, while it negatively related to the TMB in BRCA, CESC, HNSC, KIRP, LIHC, LUSC, SKCM, STAD, and UCEC (Table 1; Figure 9(a)). Furthermore, COL5A1 expression was positively related to MSI in COAD and TGCTs but negatively associated with MSI in HNSC, KIRC, SKCM, and STAD (Table 1; Figure 9(b)). Then, we assessed the correlation of COL5A1 expression with MMR genes, including MLH1, MSH2, MSH6, PMS2, and EPCAM.  Figure 9(c) illustrates the correlations between COL5A1 expression and the expression of individual MMR genes. COL5A1 expression correlated with the expression of MMR genes in most tumors, except for UCES and UVM. In addition, we used cBioPortal to investigate the correlation between COL5A1 mutations and prognosis. Patients with UCEC in the unaltered COL5A1 group experienced a shorter OS (Figure 9(d)), DSS (Figure 9(e)), and PFI (Figure 9(f)) than those in the altered group. However, patients with ESCA presenting altered COL5A1 expression experienced a shorter OS (Figure 9(g)) and PFI (Figure 9(h)).

### 3.5. Correlations of COL5A1 Methylation with Prognosis

**Figure 10:**
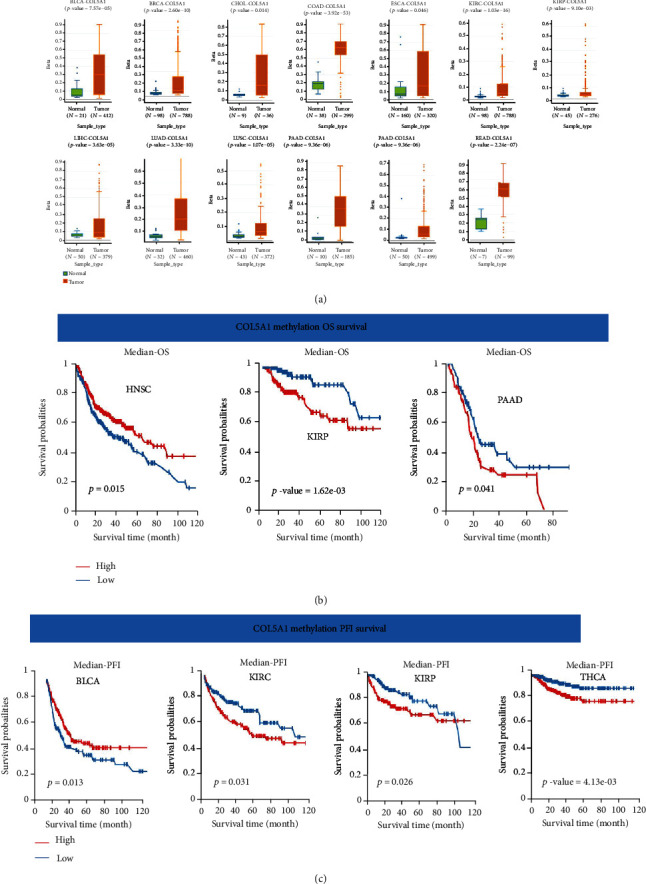
Correlations of COL5A1 with DNA methylation. (a) Differential COL5A1 methylation levels in tumor and normal tissues. Correlations between COL5A1 methylation and (b) OS and (c) PFI survival.

We further investigated the DNA methylation levels of COL5A1 in 33 cancer types. The DNA methylation of COL5A1 was increased in BLCA (*p* = 7.57*e* − 05), BRCA (*p* = 2.60*e* − 10), CHOL (*p* = 0.014), COAD (*p* = 3.92*e* − 53), ESCA (*p* = 0.046), KIRC (*p* = 1.03*e* − 16), KIRP (*p* = 9.10*e* − 03), LIHC (*p* = 3.63*e* − 05), LUAD (*p* = 3.33*e* − 10), LUSC (*p* = 1.07*e* − 05), PAAD (*p* = 9.36*e* − 06), PRAD (*p* = 1.17*e* − 05), and READ (*p* = 2.24*e* − 07) compared with normal tissues (Figure 10(a)). The DNA methylation of COL5A1 in other cancers was not significantly different from that in normal tissues. Additionally, the correlation between COL5A1 methylation and the prognosis was evaluated. Patients with HNSC presenting low COL5A1 methylation levels experienced a shorter OS than those with high COL5A1 methylation levels (*p* = 0.015) (Figure 10(b)). Nevertheless, in patients with KIRP (*p* = 1.62*e* − 03) and PAAD (*p* = 0.041), high COL5A1 methylation levels were related to a shorter OS (Figure 10(b)). High COL5A1 methylation levels were correlated to a better PFI in BLCA patients (*p* = 0.013) but linked to a poor PFI in KIRC patients (*p* = 0.031), KIRP (*p* = 0.026), and THCA (*p* = 4.13*e* − 03) (Figure 10(c)).

### 3.6. Correlations of COL5A1 Expression with TME across Cancers

**Figure 11:**
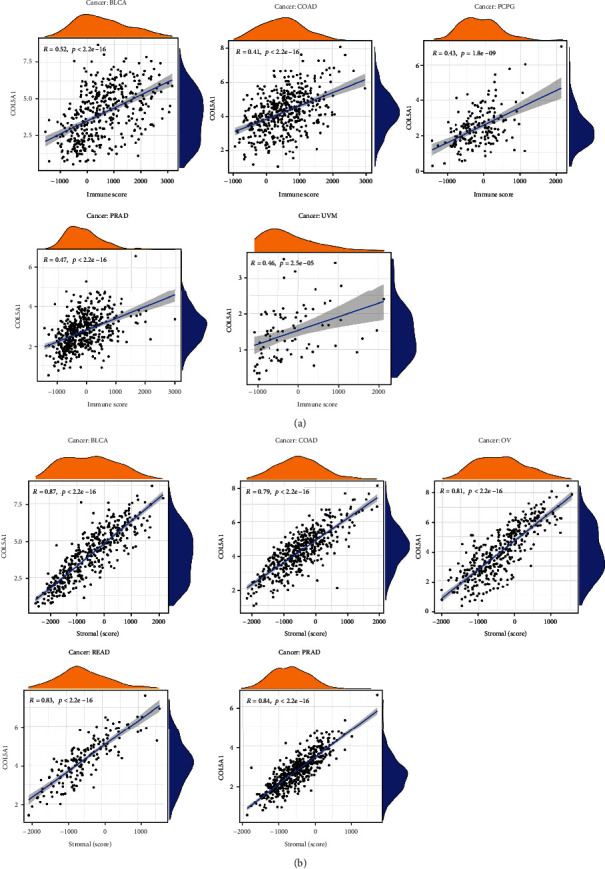
Five cancers with the highest correlation coefficients between COL5A1 expression and the TME. (a) Correlation between COL5A1 expression and stromal scores in BLCA, COAD, PCPG, PRAD, and UVM. (b) Correlation of COL5A1 expression with immune scores in BLCA, COAD, OV, READ, and PRAD.

It has been demonstrated that TME has an influential action in tumorigenesis [27], multidrug resistance, and metastasis of cancer cells [28]. Therefore, an investigation of the associations of COL5A1 expression with the TME is necessary. The ESTIMATE algorithm was employed to assess the correlations of COL5A1 expression with stromal and immune scores. The results elucidated that COL5A1 expression positively related to immune scores in 19 cancers but negatively associated with immune scores in TGCTs (Figure 11(a); Figure S4). Additionally, COL5A1 expression was positively linked to stromal scores in 30 human cancers (Figure 11(a); Figure S4). The five cancers with the highest correlation coefficients between the TME and COL5A1 expression are shown in Figure 11; data for other tumors are presented in Figure S4.

### 3.7. Association of COL5A1 Expression with the TIIC Infiltration in Human Tumors

The relationship of COL5A1 expression with the infiltration levels of 22 immunocyte subtypes was investigated. The levels of TIIC infiltration were correlated significantly with COL5A1 expression in most human tumors (Table S1). Six cancer types, including PRAD (*n* = 15), BRCA (*n* = 13), THCA (*n* = 12), BLCA (*n* = 11), KIRC (*n* = 11), GBM (*n* = 10), and KIRP (*n* = 10), showed the highest correlation between COL5A1 expression and levels of infiltrating TIIC, and the results are presented in Table 2. COL5A1 expression correlated positively with the infiltration of naive B cells in PRAD, THCA, BLCA, KIRC, and KIRP and negatively with the infiltration of memory B cells in PRAD, BRCA, BLCA, KIRC, and KIRP. Additionally, COL5A1 expression was positively correlated with the infiltration of monocytes in PRAD, BRCA, THCA, and BLCA but had a negative association with KIRC.

**Figure 12:**
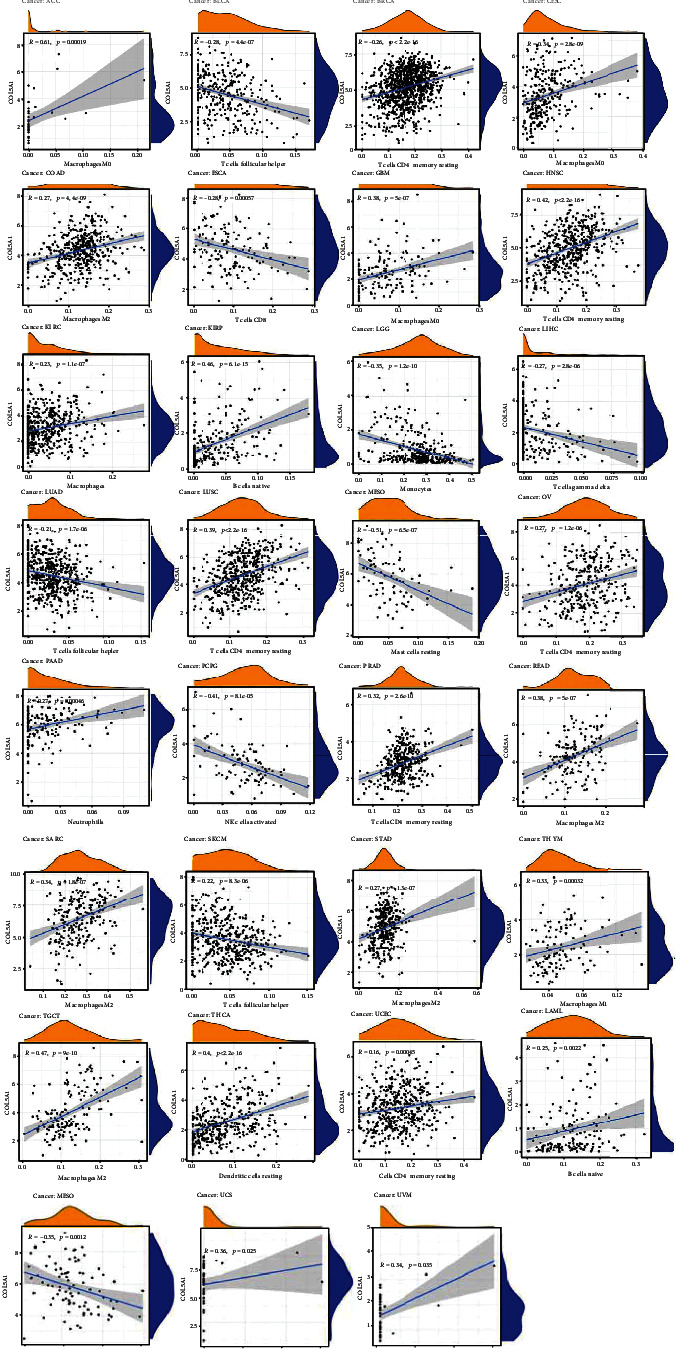
Association of COL5A1 with the degree of infiltration of different TIICs in different human tumors.

**Figure 13:**
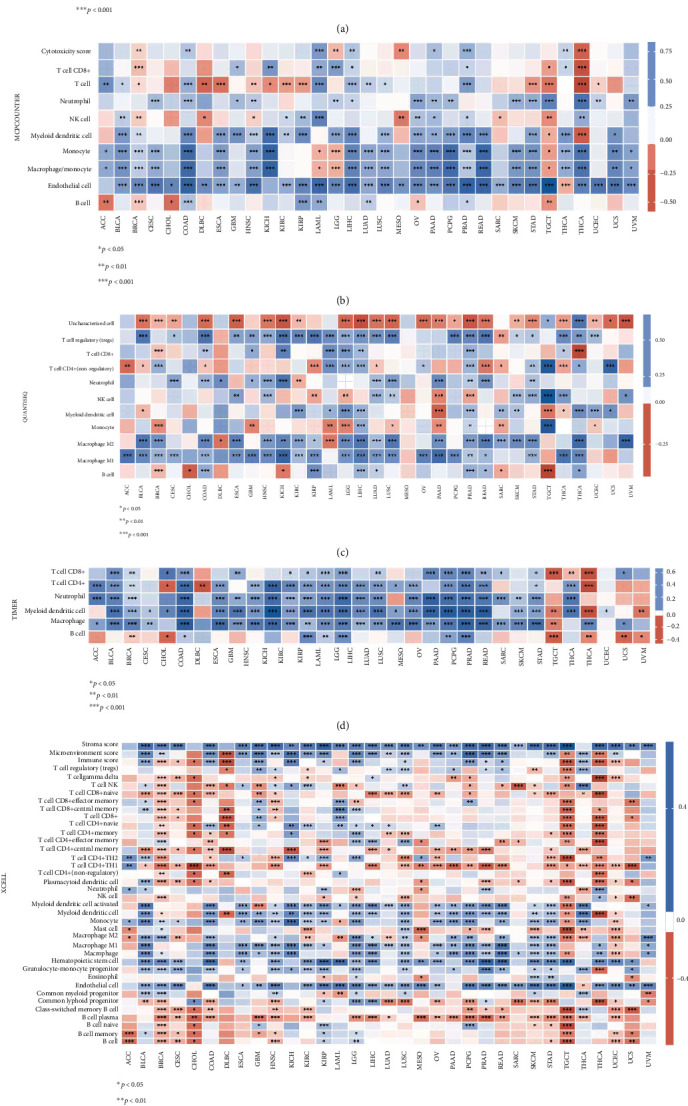
Heat map showing the correlations of COL5A1 expression with TIIC based on EPIC (a), MCP-counter (b), quanTIseq (c), TIMER (d), and xCell (e) algorithm.

Furthermore, different correlations were observed between COL5A1 expression and different subtypes of infiltrating macrophages and T cells. For example, the expression levels of COL5A1 were negatively linked to the infiltration of CD8 T cells in PRAD, BRCA, THCA, and BLCA but showed a positive relationship with the infiltrating degrees of activated memory CD4 T cells in THCA, KIRC, GBM, and KIRP (Table 2). In addition, COL5A1 expression was positively correlated with the infiltration of M0 macrophages in BRCA, BLCA, KIRC, and GBM but negatively linked to the infiltration of M2 macrophages in THCA, GBM, and KIRP. The infiltrated immune cells with the highest correlation between COL5A1 expressions in 31 cancer types are presented in Figure 12; data for other cancers are shown in Table S1. In addition, we subsequently used five algorithms, including TIMER, xCell, MCP-counter, EPIC, and quanTIseq, to confirm the correlation of COL5A1 expression with TIIC. As shown in Figures 13(a)–13(e), COL5A1 expression was correlated with TIIC in almost all cancer types.

### 3.8. Coexpression of COL5A1 with Immune-Related Genes and Pathway Enrichment Analyses in Human Tumors

**Figure 14:**
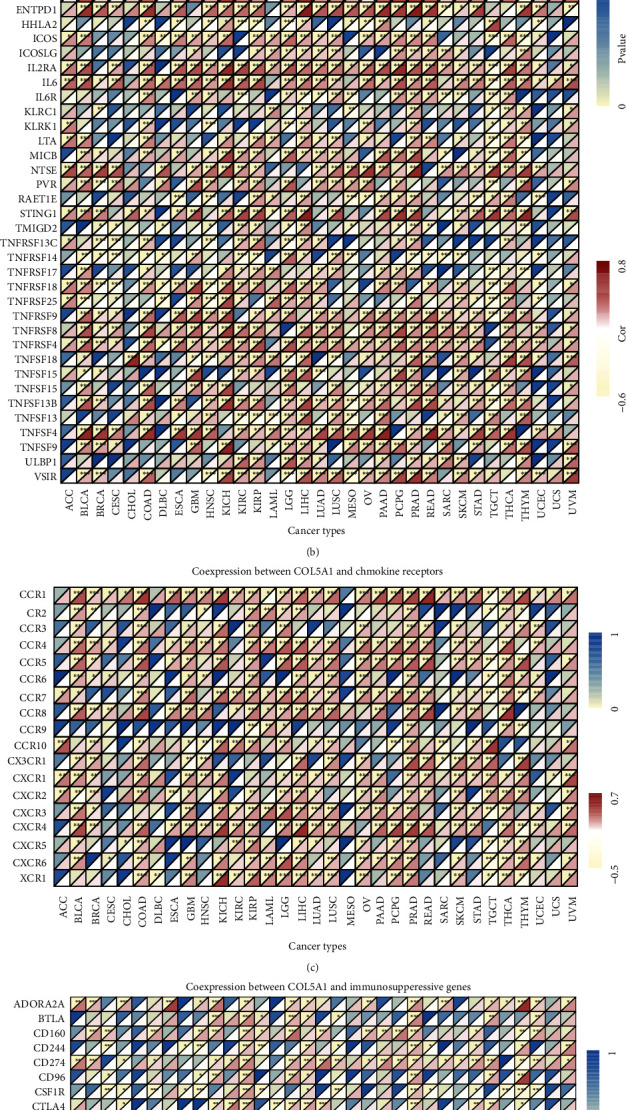
Coexpression of COL5A1 with immune-associated genes. ^∗^*p* < 0.05, ^∗∗^*p* < 0.01, and ^∗∗∗^*p* < 0.001.

Gene coexpression analyses were carried out to assess the correlations of COL5A1 expression with immune-associated genes in human tumors. The genes encoding MHC, immune activators, immune suppressors, chemokines, and chemokine receptors were investigated. The heat map demonstrated that most immune-related genes were coexpressed with COL5A1, and major immune activation, chemokine, and chemokine receptor genes exhibited a positive correlation with COL5A1 expression in major cancers (Figure 14).

**Figure 15:**
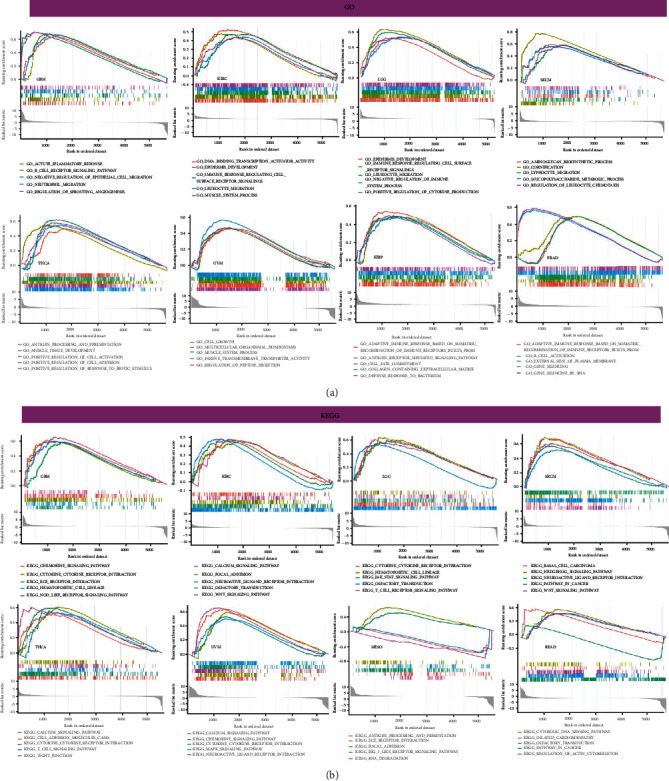
Pathway analyses of COL5A1 in different cancers. (a) GO functional annotations of COL5A1 and (b) KEGG pathway analyses of COL5A1 in various cancers.

Afterward, we carried out GO functional annotations and KEGG pathways related to COL5A1 in human tumors. As shown in Figure 15 and Figure S5, the data illustrated that COL5A1 regulated some immune-related functions in 18 cancer types (Figure 15, Figure S5). In GBM, COL5A1 expression positively regulated the acute inflammatory response, B cell receptor signaling pathway, chemokine signaling pathway, cytokine cytokine-receptor interaction, JAK-STAT pathway, and NOD-like receptor pathway, which are all immune-related signaling pathways. GO functional annotations revealed that COL5A1 positively regulated the immune response regulating cell surface receptor signaling in KIRC and LGG, and KEGG pathway analyses also showed that COL5A1 positively regulated cytokine cytokine-receptor interactions and the MAPK pathway. In addition, COL5A1 positively regulated cytokine production, negative regulation of immune system process, cytokine cytokine-receptor interaction, JAK-STAT pathway, T cell receptor pathway in LGG; positively regulated lymphocyte migration, NOD-like receptor pathway, and the WNT pathway in SKCM; positively regulated antigen processing and presentation, chemokine pathway, JAK-STAT pathway, and cytokine cytokine-receptor interaction in THCA; positively regulated cell growth, chemokine pathway, MAPK pathway, and cytokine cytokine-receptor interaction in UVM; positively regulated adaptive immune response based on somatic recombination of immune receptors built from immunoglobulin superfamily domains in KIRP and PRAD; positively regulated antigen receptor mediated pathway in KIRP; and positively regulated B cell activation in PRAD. Furthermore, the chemokine pathway and cytokine cytokine-receptor interaction were positively regulated in READ, while antigen processing and presentation and the RIG I-like receptor pathway were negatively regulated by COL5A1 in MESO. Data for other cancers are shown in Figure S5.

### 3.9. Correlation of COL5A1 with Immune Checkpoint-Related Genes and Drug Sensitivity

**Figure 16:**
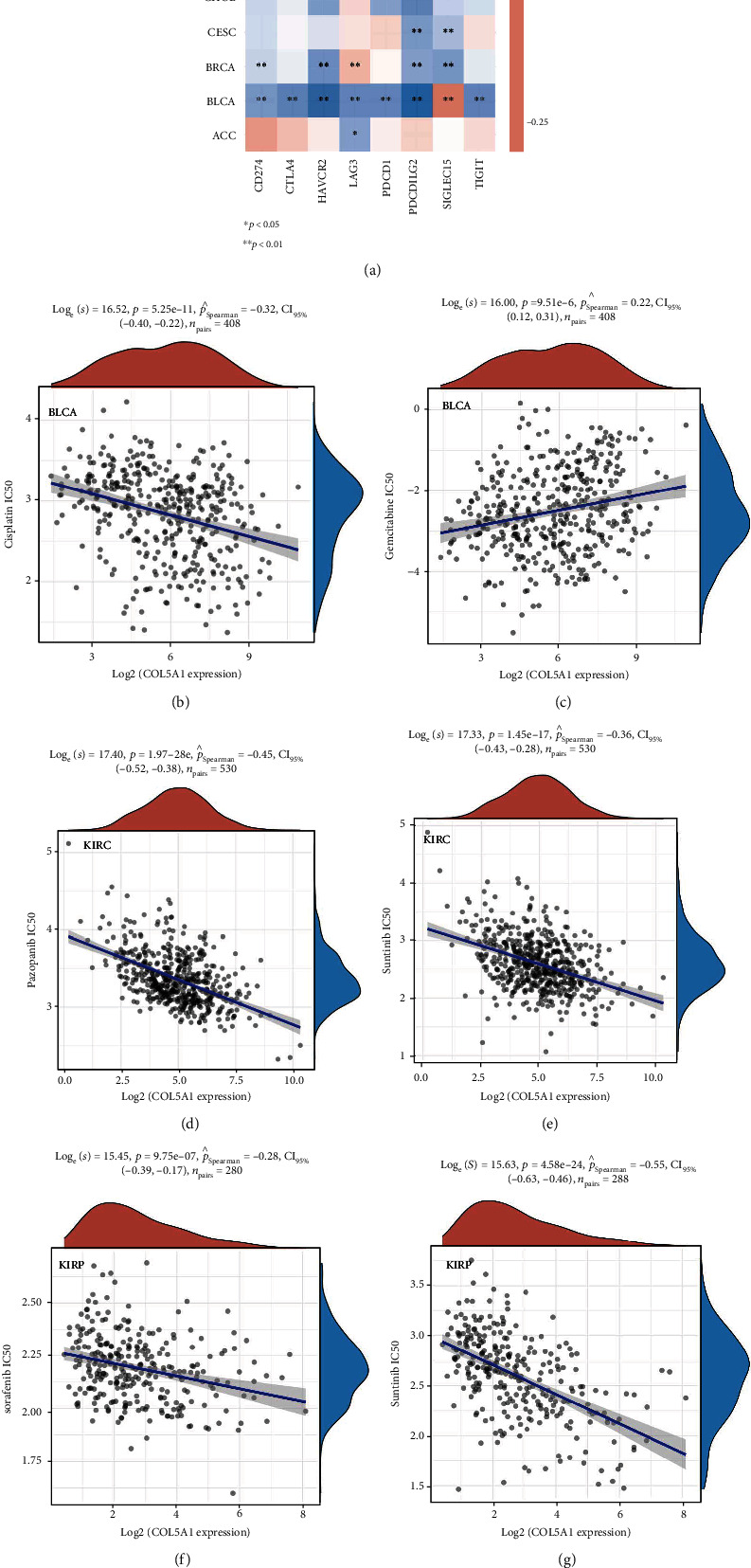
Correlation of COL5A1 with immune checkpoint-related genes and drug sensitivity. (a) Heatmap showing the correlation between the COL5A1 and immune checkpoint-related genes. (b–k) Correlation between the COL5A1 and the IC50 of chemotherapeutic drug in various tumors. ^∗^*p* < 0.05, ^∗∗^*p* < 0.01.

Immunotherapy or targeted therapy is now more and more used for a variety of tumor patients, particularly ICB therapy for patients with unsatisfactory radiotherapy and chemotherapy [20]. Therefore, it is necessary to preliminarily reveal the value of COL5A1 in predicting drug sensitivity and immunotherapy sensitivity of tumor cells through biological methods. Hence, we investigated the correlation of COL5A1 and immune checkpoints, including PDCD1, LAG3, CD274, SIGLEC15, CTLA4, TIGIT, HAVCR2, and PDCD1LG2, which were associated with response to ICB [29, 30]. The relationship between COL5A1 and immune checkpoints may be a predictor for ICB. In the cancers which had a correlation of COL5A1 with OS, including SKCM, ACC, GBM, BLCA, CESC, KIRC, LAML, KIRP, LGG, MESO, and UVM (Figure 3), the expression of COL5A1 had a correlation with immune checkpoints, especially in LGG and KIRP (Figure 16(a)). We further assessed the relationship between COL5A1 and the IC50 of chemotherapeutic drug usually used in these cancer types. We found that in BLCA, COL5A1 expression had a negative correlation between the IC50 of cisplatin, while having a positive relationship with the IC50 of gemcitabine (Figures 16(b) and 16(c)). In KIRC, the IC50 of pazopanib and sunitinib was negatively related to the expression of COL5A1 (Figures 16(d) and 16(e)). Additionally, COL5A1 expression was also negative correlated with the IC50 of sorafenib and sunitinib in KIRP (Figures 16(f) and 16(g)). In LAML, the IC50 of doxorubicin and etoposide had a positive correlation with COL5A1 expression (Figures 16(h) and 16(i)). However, the expression of COL5A1 negatively related to the IC50 of cisplatin and paclitaxel in MESO (Figures 16(j) and 16(k)). These results may have implications for cancer ICB and chemotherapy, for example, BLCA patients with high COL5A1 expression may have a more sensitive response to cisplatin treatment, which may provide accurate treatment protocols for chemotherapy.

### 3.10. Primary Validation of the Role of COL5A1 in Glioma Cells

**Figure 17:**
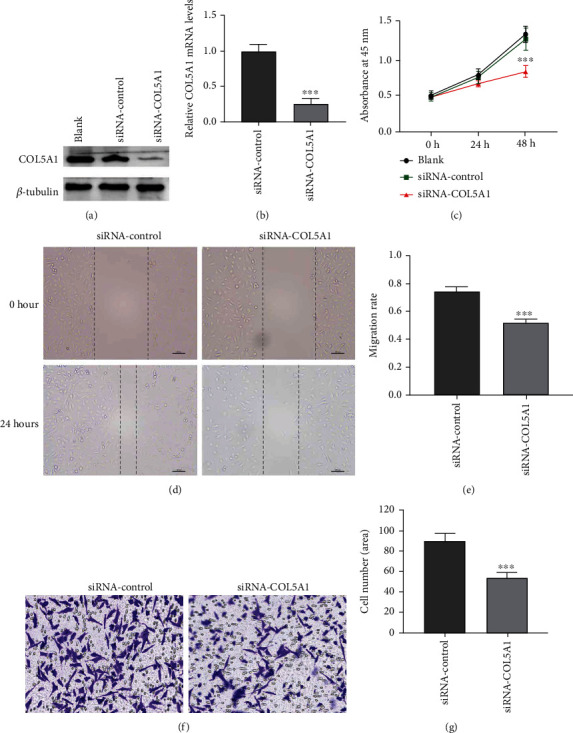
Role of COL5A1 in U251 cell proliferation, migration, and invasion. Efficiency of COL5A1 knockdown in U251 cells analyzed by (a) western blotting and (b) qRT-PCR. (d) Representative photographs of wound healing assay, bar = 100 *μ*m. (e) siRNA-COL5A1 suppressed U251 cells migration. (f, g) Transwell assay of cell migration and invasion. Amplification, ×200. ^∗∗∗^*p* < 0.001.

We found that COL5A1 was related to the prognosis of GBM and LGG patients (Figures 3(e) and 3(i)). The human glioblastoma cell line U251 was used to confirm whether COL5A1 knockdown could impact the growth of glioma cells. The results elucidated that COL5A1 knockdown decreased the proliferation of U251 cells (Figures 17(a)–17(c)). Wound healing was remarkedly inhibited by COL5A1 knockdown in U251 cells (Figures 17(d) and 17(e)). The Transwell assay demonstrated that the migration and invasion of U251 cells were significantly inhibited by COL5A1 knockdown (Figures 17(f) and 17(g)).

## 4. Discussion

As shown in the present study, COL5A1 was expressed at high levels in 13 human tumors, and IHC and western blot results confirmed this trend at the protein level. Furthermore, our findings for BRCA, ESCA, HNSC, LUAD, and STAD were similar to the results reported in previous studies [12, 31–35]. We found for the first time that COL5A1 was overexpressed in CHOL, READ, COAD, GBM, KIRC, LUSC, LIHC, and THCA. Interestingly, COL5A1 was expressed at low levels in CESC, KIRP, and UCEC compared to normal tissues. Additionally, we detected high COL5A1 expression in MESO and UCS, but insufficient expression data were available for normal tissues in TCGA. We also found that COL5A1 genetic variants existed in multiple cancer types. Additionally, DNA methylation of COL5A1 was increased in several cancers and affected the survival of patients.

KM survival analyses indicated that high COL5A1 expression was linked to a poor prognosis for patients with ACC and BLCA. Moreover, patients with BLCA presenting COL5A1 alterations showed lower disease-free survival rates [36]. Similarly, high COL5A1 expression was reported to be related to tumorigenesis, TIIC, and paclitaxel resistance in OV [16]. Chemotherapy resistance may be associated with increased tissue stiffness mediated by specific collagen cross-linking. In addition, COL5A1 was dramatically elevated in metastatic KIRC tumors compared to primary tumors [37]. In this work, high COL5A1 expression was linked to poor prognosis in patients with ACC, BLCA, GBM, KIRC, KIRP, LGG, MESO, SKCM, and UVM. Additionally, the role of COL5A1 in glioma cells was verified in vitro experiments.

Additionally, COL5A1 expression was related to age in patients with some tumors. COL5A1 was expressed at lower levels in older patients with CESC, KIRC, KIRP, LIHC, READ, and UCEC but at higher levels in older patients with BLCA, LGG, PRAD, SARC, and THYM. These results may be instructive in the selection of immunotherapy regimens for patients of different ages. Our study also revealed that COL5A1 expression was related to tumor stage in most cancers and was particularly different between stages I and IV, stages II and IV, and stages I and III. For example, in patients with CHOL, KICH, KIRP, and THCA, COL5A1 was overexpressed in stage IV tumors than in stage I tumors. A previous study revealed that COL5A1 may serve as a biomarker of the early stage of systemic sclerosis based on its autoimmune function [38]. These results suggest that COL5A1 can be served as a biomarker to determine the prognosis of a variety of cancers.

TMB has a role in providing guidance for the precise treatment of immunotherapy [39]. TMB is a biomarker linked to ICB efficacy, and higher TMB is linked to better response to ICB and prolonged OS [40]. MSI is also a biomarker linked to the ICB response [41, 42], and high-frequency MSI in COAD is a predictor for prognosis [43]. The present study illustrated that COL5A1 expression was related to the TMB in 13 tumors and MSI in 6 tumors. Thus, the expression level of COL5A1 may impact the TMB and MSI, thus affecting the patient's response to ICB. These results have a novel guiding value for immunotherapy in patients with different cancers. We assumed that among tumors showing a positive correlation between COL5A1 expression and TMB, patients with tumors presenting high COL5A1 expression and high TMB and MSI may experience a better sensitivity to ICB. We further investigated the correlation of COL5A1 with the immune checkpoints which were strongly associated with response to ICB. These results also indicated that the expression of COL5A1 may also be a predictor for the response of ICB. Additionally, the correlation of COL5A1 with the sensitivity of chemotherapeutic drugs may have implications for chemotherapy, for example, BLCA patients with high COL5A1 expression may have a more sensitive response of cisplatin treatment (Figure 16(b)).

TME can serve as predictors to assess tumor cell responses to immunotherapy [44]. In our study, COL5A1 played a critical role in the cancer immunity. This study demonstrated that COL5A1 expression was significantly and positively related to the immune component of the TME in 19 tumors and positively linked to the stromal component of the TME in 30 tumors. COL5A1 was reported as a potential target necessary for ICB in HNSC [33]. Additionally, COL5A1 expression correlated with several tumor-infiltrating cells in OV [16]. In addition, COL5A1 was reported to be negatively related to tumor purity but positively linked to immune cell infiltration, and the COL5A1-mediated cell proliferation of STAD may be mediated by effects on the TME [35]. Our study further illuminates that COL5A1 has broader oncological applicability in other tumors, and COL5A1 expression was linked to the biological progression of various TIICs. Additionally, COL5A1 is coexpressed with genes that encode MHC, immune activators, immune suppressors, chemokines, chemokine receptors, and proteins involved in the MMR. These results suggest that COL5A1 expression is related to TIIC infiltration in the tumor, affects the prognosis, and provides a novel target for improving the efficacy of immunotherapy for patients with various human tumors.

Presently, very few works have assessed the immunological action of COL5A1 in cancers, and COL5A1 is commonly presumed to be a collagen family protein (the most abundant matrix protein polymer in vertebrates) that is involved in the formation of ECM [7]. It is regarded as a key gene participating in endurance running performance [45]. COL5A1 also has a critical role in tumor development and was reported to promote the proliferation and metastasis of BRCA [46] and LUAD [47]. Notably, high COL5A1 expression is related to CD8 T cell, CD4 T cell, dendritic, macrophage, and neutrophil infiltration in STAD [35]. In addition, COL5A1 is regarded as a novel biomarker that determines sensitivity to ICB therapies in melanoma [48]. In various cancers, our enrichment analysis revealed that COL5A1 potentially influences the etiology or pathogenesis of cancer through immune-related pathways, chemokine pathways, negative regulation of immune system processes, JAK-STAT pathways, T cell receptor pathways, lymphocyte migration, NOD-like receptor pathways, antigen processing and presentation, MAPK pathways, and adaptive immune responses based on the somatic recombination of immune receptors built from immunoglobulin superfamily domains.

## 5. Conclusions

This first pan-cancer analysis of COL5A1 showed high COL5A1 expression in most tumors compared with normal tissues and revealed a correlation between COL5A1 expression and the prognosis. Based on these findings, COL5A1 may represent an independent prognostic factor for several tumors and that high COL5A1 expression levels in most tumors are linked to poor prognosis. In addition, COL5A1 expression is related to the TMB, MSI, and TIIC infiltration in some human tumors. COL5A1 may serve as a predictor for chemotherapy and immune-based ICB.

## Figures and Tables

**Table 1:** Correlation between COL5A1 expression and TMB and MSI. ^∗^*p* < 0.05, ^∗∗^*p* < 0.01, and ^∗∗∗^*p* < 0.001.

TMB			MSI		
Cancer type	Cor	*p* value	Cancer type	Cor	*p* value
ACC	0.43733147	^∗∗∗^/5.58*e*-05	COAD	0.14783620	^∗∗^/0.002193
BRCA	-0.07340527	^∗^/0.02196037	HNSC	-0.15164437	^∗∗∗^/0.000703
CESC	-0.12802692	^∗^/0.03042103	KIRC	-0.14245300	^∗∗^/0.00903
HNSC	-0.21248963	^∗∗∗^/0.00000198	SKCM	-0.13520964	^∗∗^/0.003382
KIRP	-0.25583301	^∗∗∗^/0.00001571	STAD	-0.10750443	^∗^/0.0377
LAML	0.34680128	^∗∗^/0.00536082	TGCT	0.17807180	^∗^/0.029247
LGG	0.25972873	^∗∗∗^/3.62*e* − 09			
LIHC	-0.31014868	^∗∗∗^/1.91692*e* − 09			
LUSC	-0.13299831	^∗∗^/0.00324439			
SKCM	-0.11416702	^∗^/0.01376592			
STAD	-0.12401527	^∗^/0.01730668			
THYM	0.51906320	^∗∗∗^/2.03*e* − 09			
UCEC	-0.13110071	^∗∗^/0.00261476			

**Table 2:** Relationship between COL5A1 expression and immune cell infiltration in different cancers. ^∗^*p* < 0.05, ^∗∗^*p* < 0.01, and ^∗∗∗^*p* < 0.001.

Cancer type	PRAD *p* value/Cor	BRCA *p* value/Cor	THCA *p* value/Cor	BLCA *p* value/Cor	KIRC *p* value/Cor	GBM *p* value/Cor	KIRP *p* value/Cor
B cells naive	^∗∗∗^/0.24		^∗^/0.10	^∗^/0.14	^∗∗∗^/0.23		^∗∗∗^/0.46
B cells memory	^∗∗^/-0.17	^∗∗^/-0.09		^∗∗∗^/-0.23	^∗∗^/-0.14		^∗∗∗^/-0.31
Plasma cells	^∗∗∗^/-0.22						^∗∗∗^/0.27
T cells CD8	^∗∗∗^/-0.28	^∗∗∗^/-0.19	^∗∗∗^/-0.23	^∗∗^/-0.17			
T cells CD4 naive							
T cells CD4 memory resting	^∗∗∗^/0.32	^∗∗∗^/0.26		^∗∗^/0.16			
T cells CD4 memory activated	^∗^/-0.10	^∗∗∗^/-0.19	^∗^/0.10		^∗∗∗^/0.16	^∗∗∗^/0.29	^∗∗∗^/0.28
T cells follicular helper	^∗∗∗^/-0.23	^∗∗∗^/-0.20	^∗^/0.10	^∗∗∗^/-0.28	^∗∗∗^/-0.21	^∗∗∗^/-0.28	^∗∗^/0.19
T cells regulatory (Tregs)	^∗∗∗^/0.18	^∗^/0.07	^∗∗∗^/0.18		^∗∗^/-0.09		^∗^/0.15
T cells gamma delta		^∗∗^/-0.09			^∗∗^/-0.13		
NK cells resting			^∗∗^/-0.16			^∗∗∗^/0.27	
NK cells activated	^∗^/-0.10				^∗∗∗^/-0.18	^∗∗^/-0.21	
Monocytes	^∗∗^/0.16	^∗^/0.07	^∗∗∗^/0.18	^∗^/0.13	^∗∗^/-0.13		
Macrophages M0	^∗∗∗^/-0.21	^∗^/0.07	^∗∗∗^/-0.18	^∗∗∗^/0.22	^∗∗∗^/0.23	^∗∗∗^/0.38	
Macrophages M1	^∗^/-0.11	^∗∗∗^/-0.13	^∗^/0.11			^∗^/-0.19	^∗∗∗^/0.35
Macrophages M2	^∗∗^/0.14		^∗∗∗^/-0.25	^∗∗∗^/0.27	^∗^/0.09	^∗^/-0.19	^∗∗∗^/-0.32
Dendritic cells resting	^∗∗∗^/0.23	^∗∗∗^/0.11	^∗∗∗^/0.40				^∗∗∗^/0.24
Dendritic cells activated	^∗∗∗^/0.23		^∗∗∗^/0.18	^∗∗∗^/-0.23	^∗∗∗^/-0.23		
Mast cells resting		^∗∗∗^/0.23		^∗∗^/0.14		^∗∗^/-0.20	^∗^/-0.13
Mast cells activated						^∗^/0.15	
Eosinophils							
Neutrophils		^∗∗^/0.09		^∗∗^/0.17		^∗∗∗^/0.27	

## Data Availability

The datasets generated and/or analyzed during the current study are available from the corresponding authors upon reasonable request in compliance with ethical standards.

## Supplementary Materials

Supplementary Materials Table S1: the infiltration of TIIC correlated with COL5A1 expression in various cancer types. Figure S1: representative clinicopathological photographs of the tumor and paracancerous tissues. Figure S2: correlation between age and COL5A1 expression in patients with various cancer types. Figure S3: association between COL5A1 expression and the tumor stage. Figure S4: correlation between COL5A1 expression and stromal or immune scores. Figure S5: GO and KEGG pathway analyses of COL5A1 in various tumors.

## References

[1] Bray F., Ferlay J., Soerjomataram I., Siegel R. L., Torre L. A., Jemal A. (2018). Global cancer statistics 2018: GLOBOCAN estimates of incidence and mortality worldwide for 36 cancers in 185 countries. *CA: a Cancer Journal for Clinicians*.

[2] Wang X. Y., Sun N., Meng X. Q., Chen M., Jiang C. L., Cai J. Q. (2020). Review of clinical nerve repair strategies for neurorestoration of central nervous system tumor damage. *Journal of Neurorestoratology*.

[3] Deng Z., Yu H., Wang N. (2019). Impact of preoperative Karnofsky Performance Scale (KPS) and American Society of Anesthesiologists (ASA) scores on perioperative complications in patients with recurrent glioma undergoing repeated operation. *Journal of Neurorestoratology*.

[4] Pardoll D. M. (2012). The blockade of immune checkpoints in cancer immunotherapy. *Nature Reviews. Cancer*.

[5] Blum A., Wang P., Zenklusen J. C. (2018). SnapShot: TCGA-analyzed tumors. *Cell*.

[6] Hu X., Zhu H., Zhang X., He X., Xu X. (2021). Comprehensive analysis of pan-cancer reveals potential of ASF1B as a prognostic and immunological biomarker. *Cancer Medicine*.

[7] An F., Zhang Z., Xia M., Xing L. (2015). Subpath analysis of each subtype of head and neck cancer based on the regulatory relationship between miRNAs and biological pathways. *Oncology Reports*.

[8] Jiang M., Ren L., Chen Y., Wang H., Wu H., Cheng S. (2021). Identification of a hypoxia-related signature for predicting prognosis and the immune microenvironment in bladder cancer. *Frontiers in Molecular Biosciences*.

[9] Tang X. L., Wang Z., Zhu Y. Y. (2020). Hypoxia-activated ROS burst liposomes boosted by local mild hyperthermia for photo/chemodynamic therapy. *Journal of Controlled Release*.

[10] Martins Cavaco A. C., Dâmaso S., Casimiro S., Costa L. (2020). Collagen biology making inroads into prognosis and treatment of cancer progression and metastasis. *Cancer Metastasis Reviews*.

[11] Wenstrup R. J., Florer J. B., Brunskill E. W., Bell S. M., Chervoneva I., Birk D. E. (2004). Type V Collagen Controls the Initiation of Collagen Fibril Assembly. *The Journal of Biological Chemistry*.

[12] Wu M., Sun Q., Mo C. H. (2019). Prospective molecular mechanism of COL5A1 in breast cancer based on a microarray, RNA sequencing and immunohistochemistry. *Oncology Reports*.

[13] Garcia J. M., Stillings S. A., Leclerc J. L. (2017). Role of interleukin-10 in acute brain injuries. *Frontiers in Neurology*.

[14] Chai F., Liang Y., Zhang F., Wang M., Zhong L., Jiang J. (2016). Systematically identify key genes in inflammatory and non-inflammatory breast cancer. *Gene*.

[15] Zhao X., Cai H., Wang X., Ma L. (2016). Discovery of signature genes in gastric cancer associated with prognosis. *Neoplasma*.

[16] Zhang J., Zhang J., Wang F. (2021). Overexpressed COL5A1 is correlated with tumor progression, paclitaxel resistance, and tumor-infiltrating immune cells in ovarian cancer. *Journal of Cellular Physiology*.

[17] Carlsten M., Järås M. (2019). Natural killer cells in myeloid malignancies: immune surveillance, NK cell dysfunction, and pharmacological opportunities to bolster the endogenous NK cells. *Frontiers in Immunology*.

[18] Zhu H., Hu X., Gu L. (2021). TUBA1C is a prognostic marker in low-grade glioma and correlates with immune cell infiltration in the tumor microenvironment. *Frontiers in Genetics*.

[19] Rhodes D. R., Yu J., Shanker K. (2004). *ONCOMINE*: A Cancer Microarray Database and Integrated Data-Mining Platform. *Neoplasia*.

[20] Zhu H., Hu X., Ye Y. (2021). Pan-cancer analysis of PIMREG as a biomarker for the prognostic and immunological role. *Frontiers in Genetics*.

[21] Diboun I., Wernisch L., Orengo C. A., Koltzenburg M. (2006). Microarray analysis after RNA amplification can detect pronounced differences in gene expression using limma. *BMC Genomics*.

[22] Newman A. M., Liu C. L., Green M. R. (2015). Robust enumeration of cell subsets from tissue expression profiles. *Nature Methods*.

[23] Lu X., Jiang L., Zhang L. (2019). Immune signature-based subtypes of cervical squamous cell carcinoma tightly associated with human papillomavirus type 16 expression, molecular features, and clinical outcome. *Neoplasia*.

[24] Feng G., Ma H. M., Huang H. B. (2019). <p>Overexpression of COL5A1 promotes tumor progression and metastasis and correlates with poor survival of patients with clear cell renal cell carcinoma</p>. *Cancer Management and Research*.

[25] Guo X. D., Ji J., Xue T. F. (2020). FTY720 exerts anti-glioma effects by regulating the glioma microenvironment through increased CXCR4 internalization by glioma-associated microglia. *Frontiers in Immunology*.

[26] Pu B., Zhang X., Yan T. (2021). MICAL2 promotes proliferation and migration of glioblastoma cells through TGF-*β*/p-Smad2/EMT-like signaling Pathway. *Oncology*.

[27] Fane M., Weeraratna A. T. (2020). How the ageing microenvironment influences tumour progression. *Nature Reviews. Cancer*.

[28] Gasser S., Lim L. H. K., Cheung F. S. G. (2017). The role of the tumour microenvironment in immunotherapy. *Endocrine-Related Cancer*.

[29] Nebhan C. A., Johnson D. B. (2020). Predictive biomarkers of response to immune checkpoint inhibitors in melanoma. *Expert Review of Anticancer Therapy*.

[30] Marwitz S., Scheufele S., Perner S., Reck M., Ammerpohl O., Goldmann T. (2017). Epigenetic modifications of the immune-checkpoint genes CTLA4 and PDCD1 in non-small cell lung cancer results in increased expression. *Epigenetics*.

[31] Ren W., Zhang Y., Zhang L., Lin Q., Zhang J., Xu G. (2018). Overexpression of collagen type V *α*1 chain in human breast invasive ductal carcinoma is mediated by TGF-*β*1. *International Journal of Oncology*.

[32] Chen F. F., Zhang S. R., Peng H., Chen Y. Z., Cui X. B. (2019). Integrative genomics analysis of hub genes and their relationship with prognosis and signaling pathways in esophageal squamous cell carcinoma. *Molecular Medicine Reports*.

[33] Chen Y., Li Z. Y., Zhou G. Q., Sun Y. (2021). An immune-related gene prognostic index for head and neck squamous cell carcinoma. *Clinical Cancer Research*.

[34] Wu M., Xu L., Wang Y. (2018). S100A8/A9 induces microglia activation and promotes the apoptosis of oligodendrocyte precursor cells by activating the NF-*κ*B signaling pathway. *Brain Research Bulletin*.

[35] Wei Z., Chen L., Meng L., Han W., Huang L., Xu A. (2020). LncRNA HOTAIR promotes the growth and metastasis of gastric cancer by sponging miR-1277-5p and upregulating COL5A1. *Gastric Cancer*.

[36] Guo C., Shao T., Wei D. (2020). Bioinformatic identification of potential hub genes in muscle-invasive bladder urothelial carcinoma. *Cell Transplantation*.

[37] Gao S., Yan L., Zhang H., Fan X., Jiao X., Shao F. (2021). Identification of a metastasis-associated gene signature of clear cell renal cell carcinoma. *Frontiers in Genetics*.

[38] Velosa A. P. P., Brito L., de Jesus Queiroz Z. A. (2021). Identification of autoimmunity to peptides of collagen V *α*1 chain as newly biomarkers of early stage of systemic sclerosis. *Frontiers in Immunology*.

[39] Fumet J. D., Truntzer C., Yarchoan M., Ghiringhelli F. (2020). Tumour mutational burden as a biomarker for immunotherapy: current data and emerging concepts. *European Journal of Cancer*.

[40] Samstein R. M., Lee C. H., Shoushtari A. N. (2019). Tumor mutational load predicts survival after immunotherapy across multiple cancer types. *Nature Genetics*.

[41] Lee D. W., Han S. W., Bae J. M. (2019). Tumor mutation burden and prognosis in patients with colorectal cancer treated with adjuvant fluoropyrimidine and oxaliplatin. *Clinical Cancer Research*.

[42] Boland C. R., Goel A. (2010). Microsatellite instability in colorectal cancer. *Gastroenterology*.

[43] Gryfe R., Kim H., Hsieh E. T. (2000). Tumor microsatellite instability and clinical outcome in young patients with colorectal cancer. *The New England Journal of Medicine*.

[44] Wu T., Dai Y. (2017). Tumor microenvironment and therapeutic response. *Cancer Letters*.

[45] Posthumus M., Schwellnus M. P., Collins M. (2011). The COL5A1 Gene. *Medicine and Science in Sports and Exercise*.

[46] Zhao B., Song X., Guan H. (2020). CircACAP2 promotes breast cancer proliferation and metastasis by targeting miR-29a/b-3p-COL5A1 axis. *Life Sciences*.

[47] Liu W., Wei H., Gao Z. (2018). _COL5A1_ may contribute the metastasis of lung adenocarcinoma. *Gene*.

[48] Chen H., Yang M., Wang Q., Song F., Li X., Chen K. (2019). The new identified biomarkers determine sensitivity to immune check-point blockade therapies in melanoma. *Oncoimmunology*.

