# Supplementary material for: The Hypoxia-Related Gene COL5A1 Is a Prognostic and Immunological Biomarker for Multiple Human Tumors
Source: Oxid Med Cell Longev. 2022 Jan 17;2022:6419695. doi: 10.1155/2022/6419695 (PMC8786464; doi:10.1155/2022/6419695)
Supplement: Supplementary Materials — Table S1: the infiltration of TIIC correlated with COL5A1 expression in various cancer types. Figure S1: representative clinicopathological photographs of the tumor and paracancerous tissues. Figure S2: correlation between age and COL5A1 expression in patients with various cancer types. Figure S3: association between COL5A1 expression and the tumor stage. Figure S4: correlation between COL5A1 expression and stromal or immune scores. Figure S5: GO and KEGG pathway analyses of COL5A1 in various tumors. [file 6419695.f1.zip › Figure S3 (1).pdf]

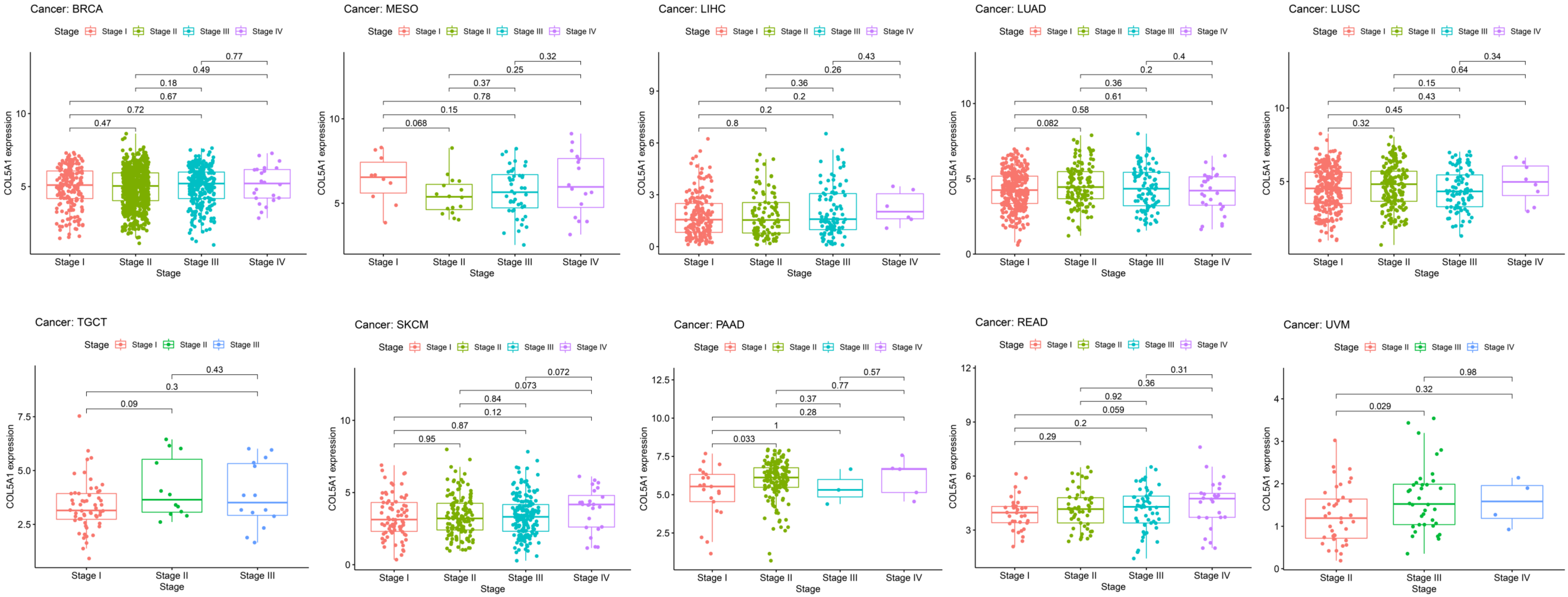

Cancer: BRCA
Stage Stage I Stage II Stage III Stage IV
0.77
0.49
0.18
0.67
0.72
0.47
COL5A1 expression
Stage I Stage II Stage III Stage IV
Stage
Cancer: MESO
Stage Stage I Stage II Stage III Stage IV
0.32
0.25
0.37
0.78
0.15
0.068
COL5A1 expression
Stage I Stage II Stage III Stage IV
Stage
Cancer: LIHC
Stage Stage I Stage II Stage III Stage IV
0.43
0.26
0.36
0.2
0.2
0.8
COL5A1 expression
Stage I Stage II Stage III Stage IV
Stage
Cancer: LUAD
Stage Stage I Stage II Stage III Stage IV
0.4
0.2
0.36
0.61
0.58
0.082
COL5A1 expression
Stage I Stage II Stage III Stage IV
Stage
Cancer: LUSC
Stage Stage I Stage II Stage III Stage IV
0.34
0.64
0.15
0.43
0.45
0.32
COL5A1 expression
Stage I Stage II Stage III Stage IV
Stage
Cancer: TGCT
Stage Stage I Stage II Stage III
0.43
0.3
0.09
COL5A1 expression
Stage I Stage II Stage III
Stage
Cancer: SKCM
Stage Stage I Stage II Stage III Stage IV
0.072
0.073
0.84
0.12
0.87
0.95
COL5A1 expression
Stage I Stage II Stage III Stage IV
Stage
Cancer: PAAD
Stage Stage I Stage II Stage III Stage IV
0.57
0.77
0.37
0.28
1
0.033
COL5A1 expression
Stage I Stage II Stage III Stage IV
Stage
Cancer: READ
Stage Stage I Stage II Stage III Stage IV
0.31
0.36
0.92
0.059
0.2
0.29
COL5A1 expression
Stage I Stage II Stage III Stage IV
Stage
Cancer: UVM
Stage Stage II Stage III Stage IV
0.98
0.32
0.029
COL5A1 expression
Stage II Stage III Stage IV
Stage
