# Supplementary figures and images for: The Hypoxia-Related Gene COL5A1 Is a Prognostic and Immunological Biomarker for Multiple Human Tumors
Source: Oxid Med Cell Longev. 2022 Jan 17;2022:6419695. doi: 10.1155/2022/6419695 (PMC8786464; doi:10.1155/2022/6419695)

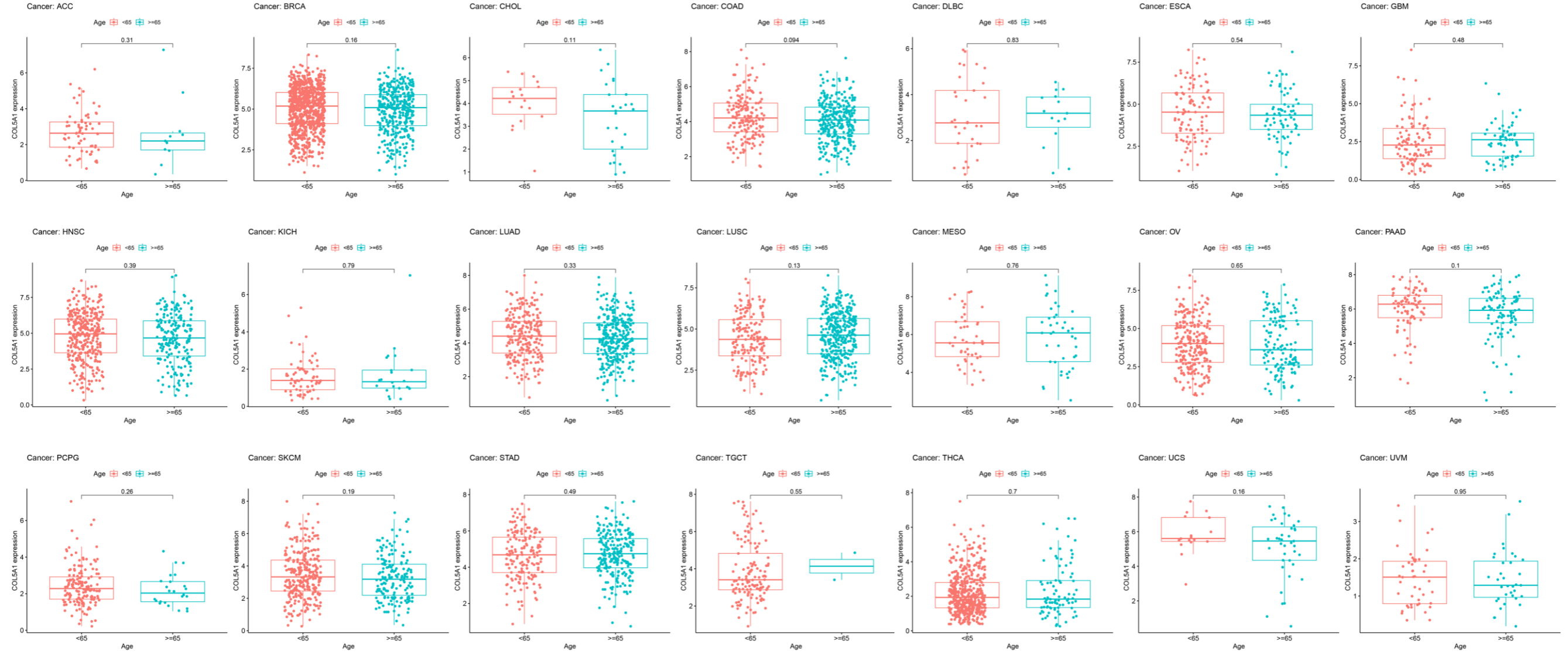

Supplement: Supplementary Materials — Table S1: the infiltration of TIIC correlated with COL5A1 expression in various cancer types. Figure S1: representative clinicopathological photographs of the tumor and paracancerous tissues. Figure S2: correlation between age and COL5A1 expression in patients with various cancer types. Figure S3: association between COL5A1 expression and the tumor stage. Figure S4: correlation between COL5A1 expression and stromal or immune scores. Figure S5: GO and KEGG pathway analyses of COL5A1 in various tumors. [file 6419695.f1.zip › Figure S2 (1).pdf]

Immune score

Stromal score

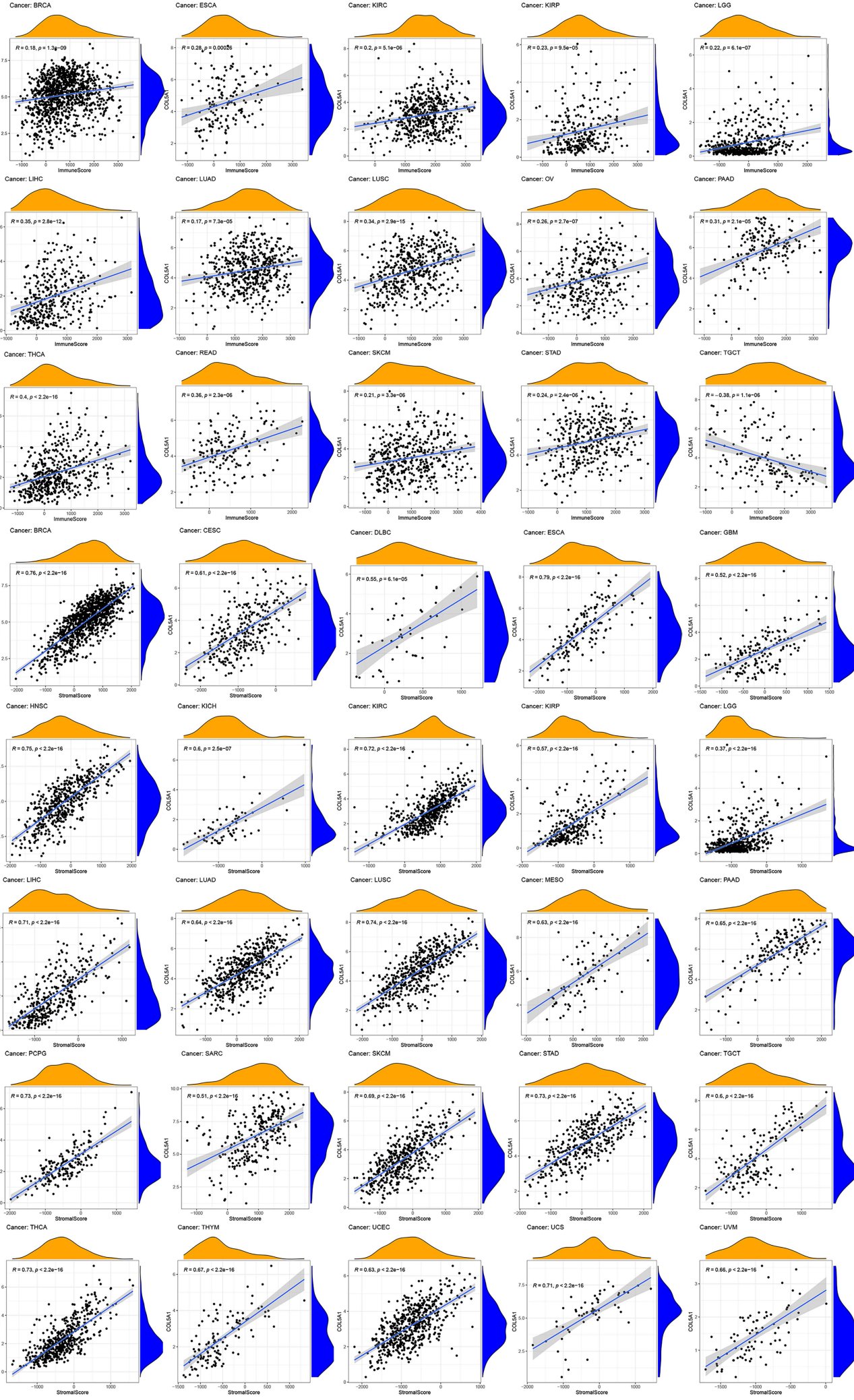

Supplement: Supplementary Materials — Table S1: the infiltration of TIIC correlated with COL5A1 expression in various cancer types. Figure S1: representative clinicopathological photographs of the tumor and paracancerous tissues. Figure S2: correlation between age and COL5A1 expression in patients with various cancer types. Figure S3: association between COL5A1 expression and the tumor stage. Figure S4: correlation between COL5A1 expression and stromal or immune scores. Figure S5: GO and KEGG pathway analyses of COL5A1 in various tumors. [file 6419695.f1.zip › Figure S4 (1).pdf]

# GO

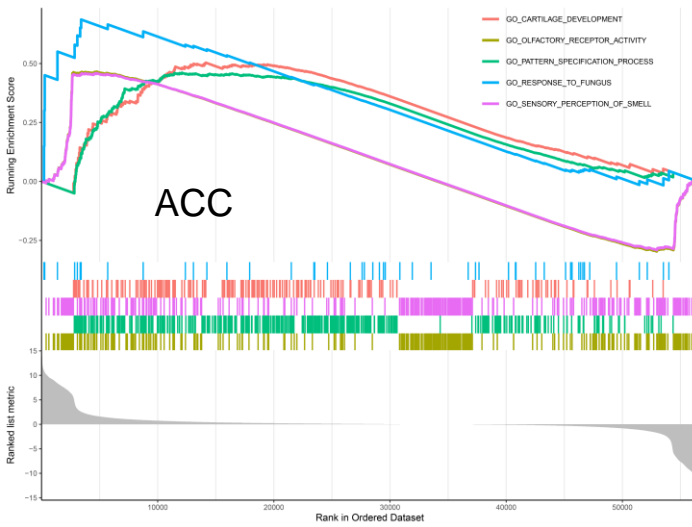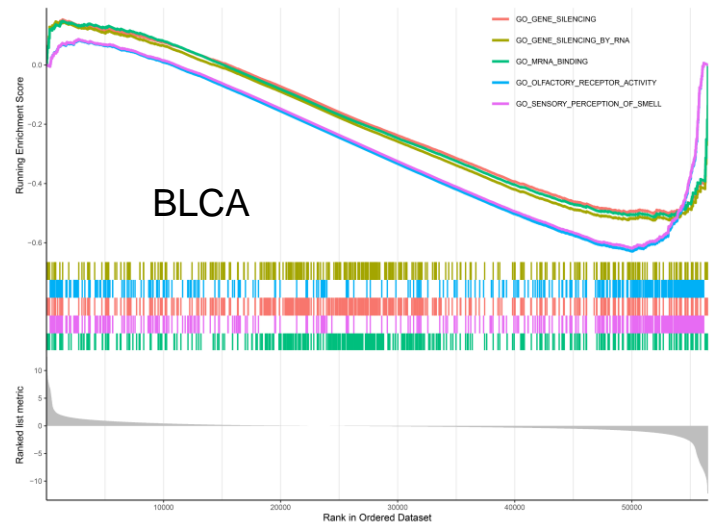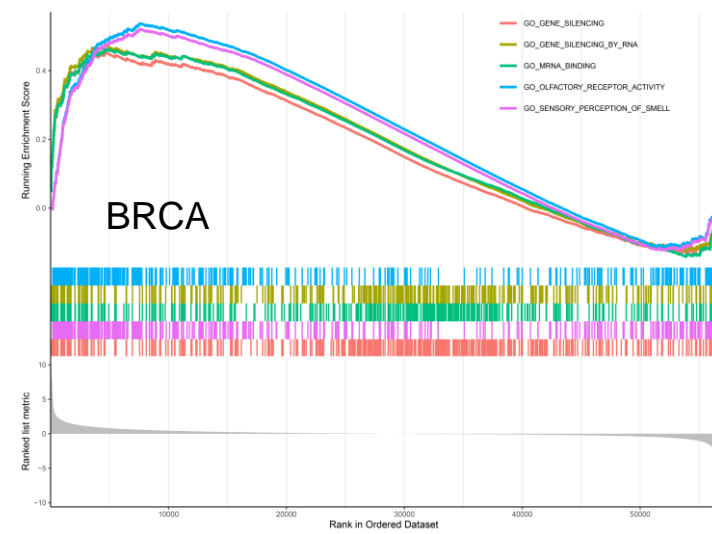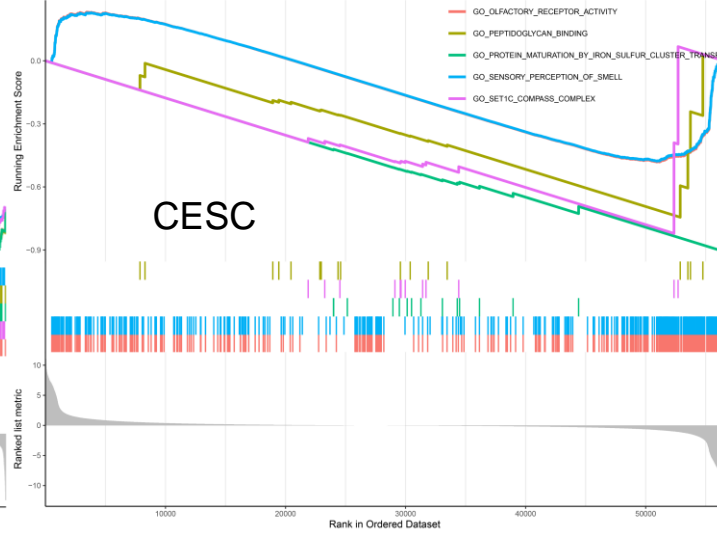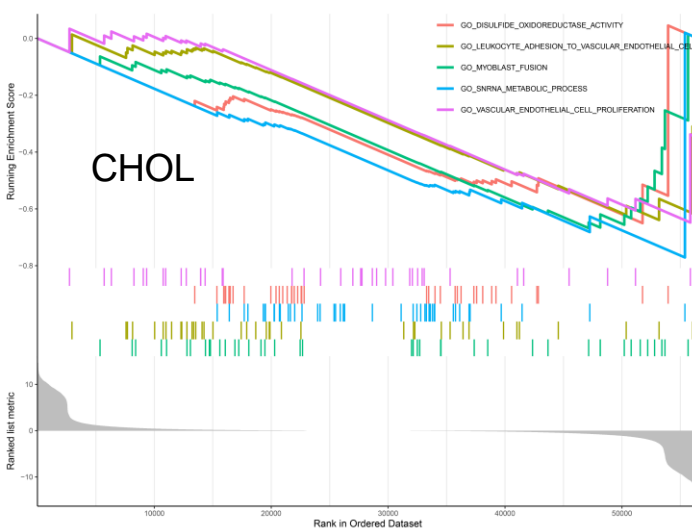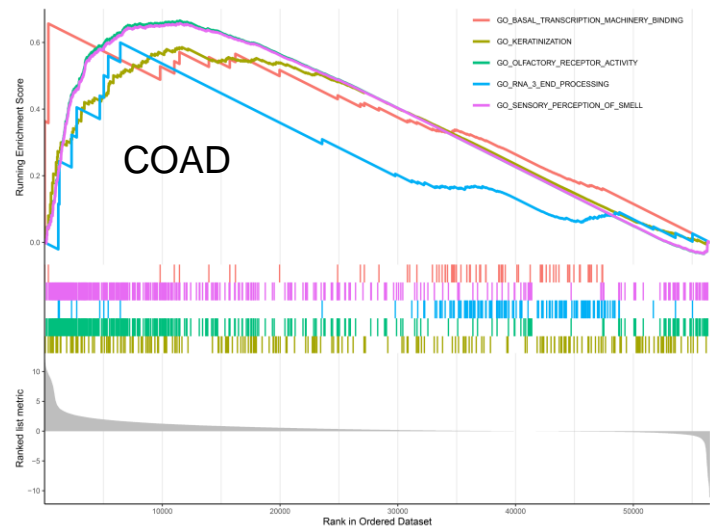

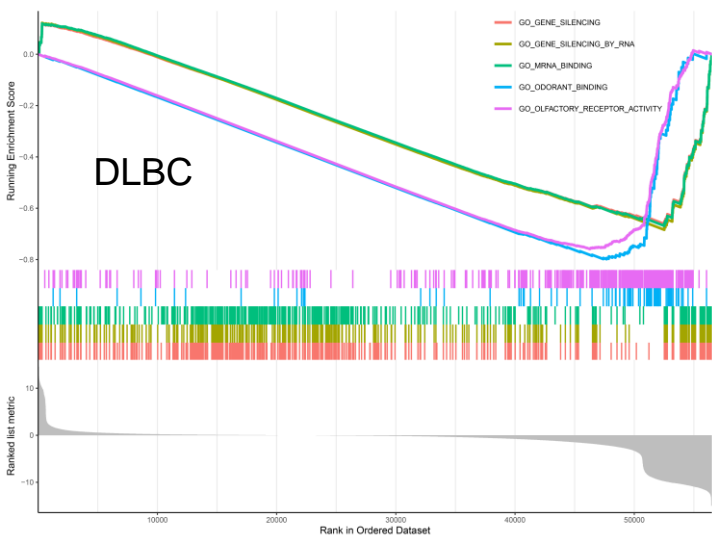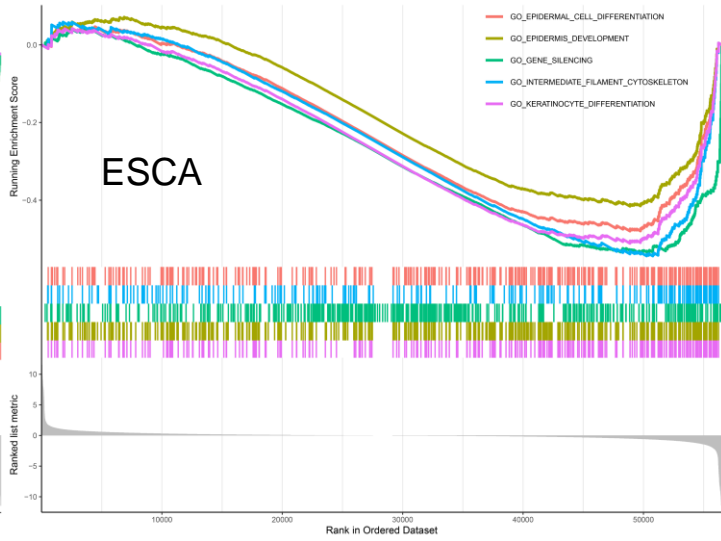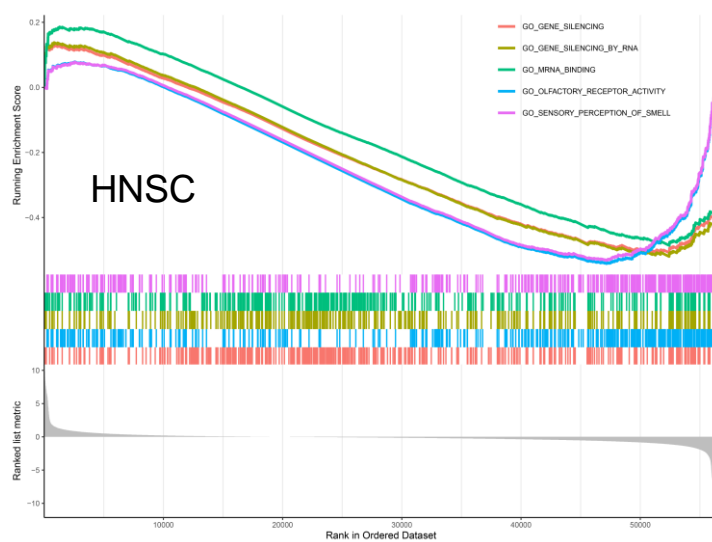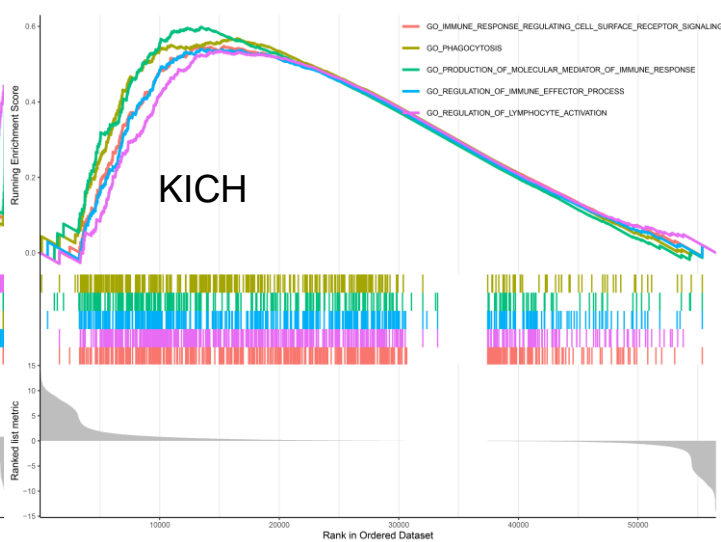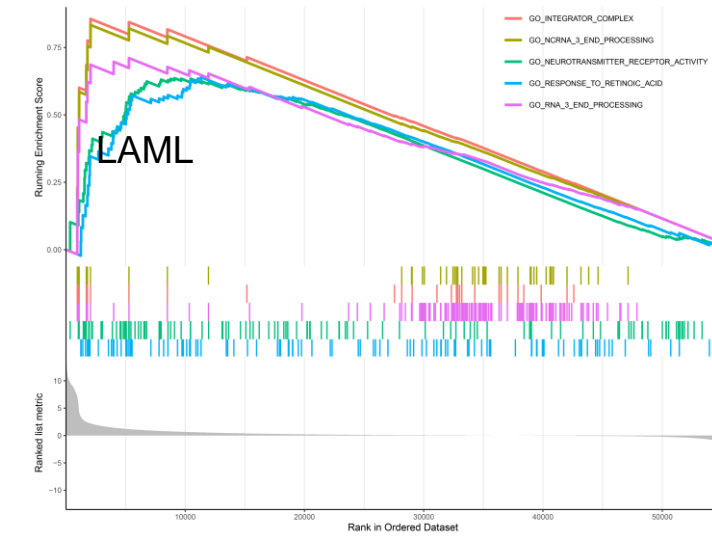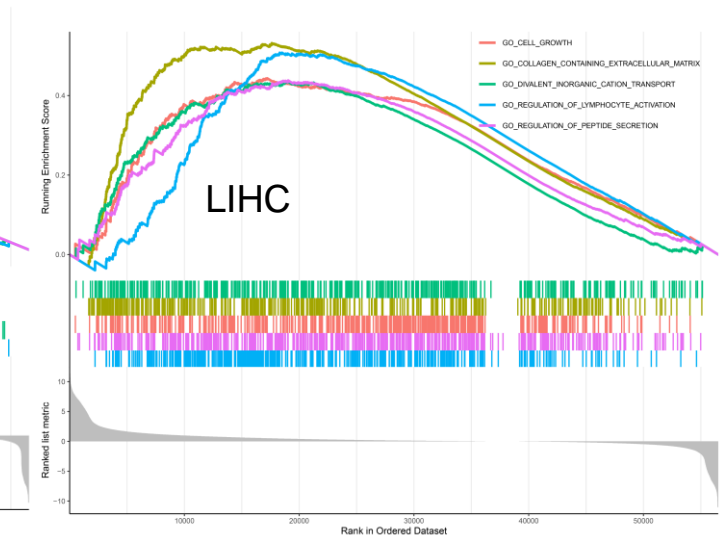

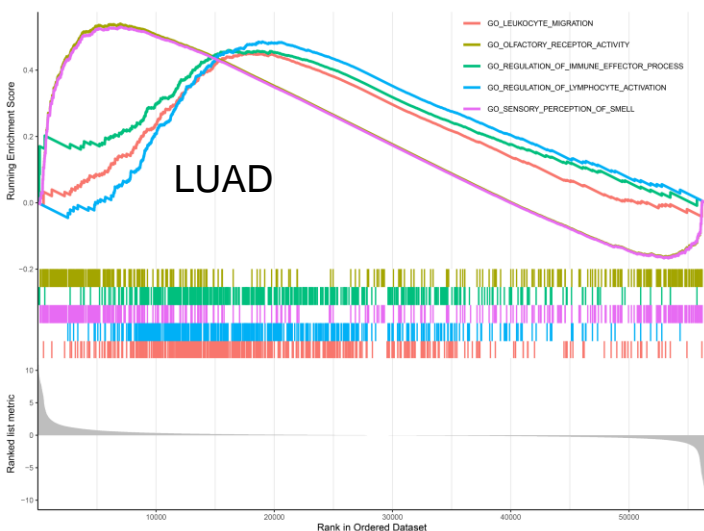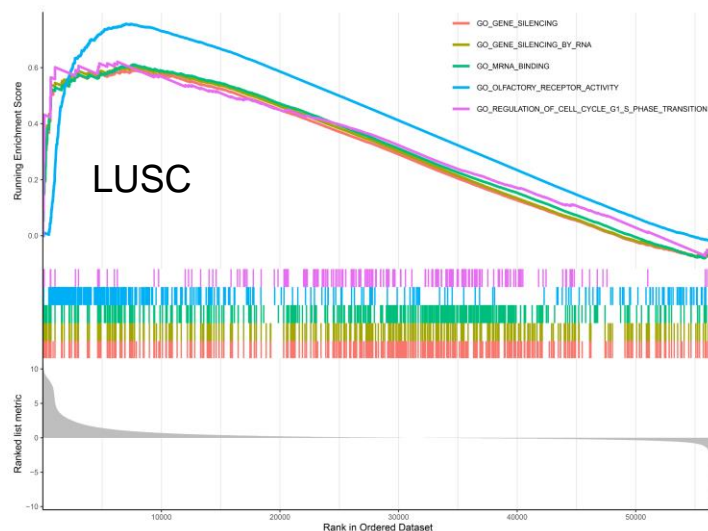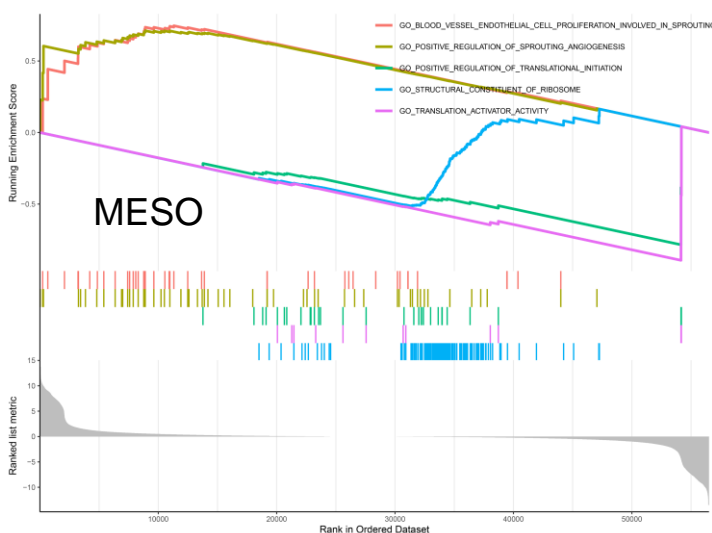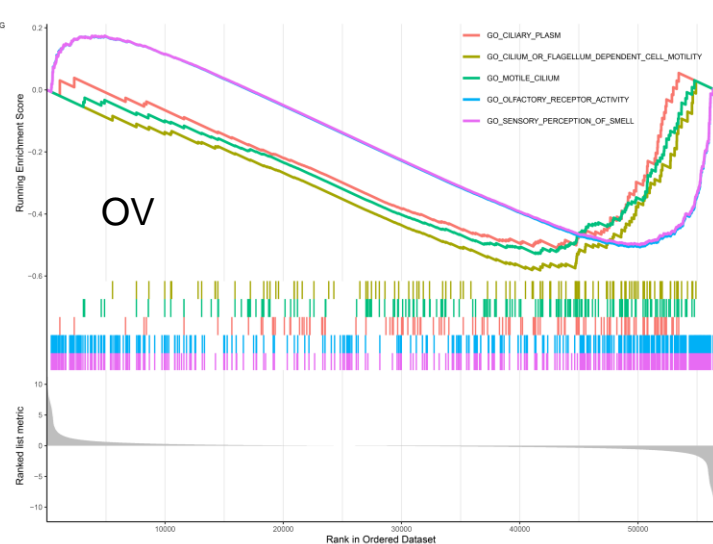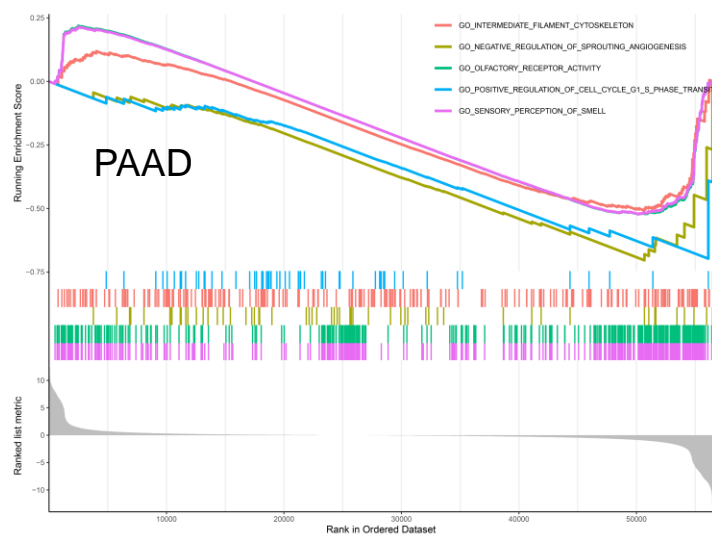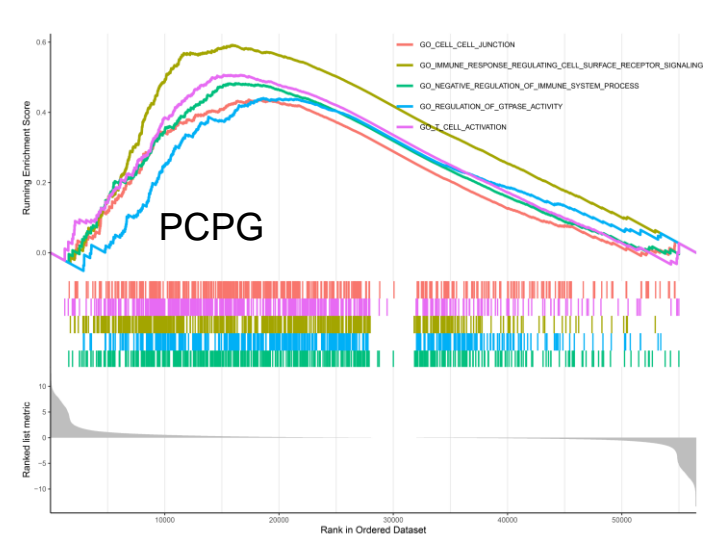

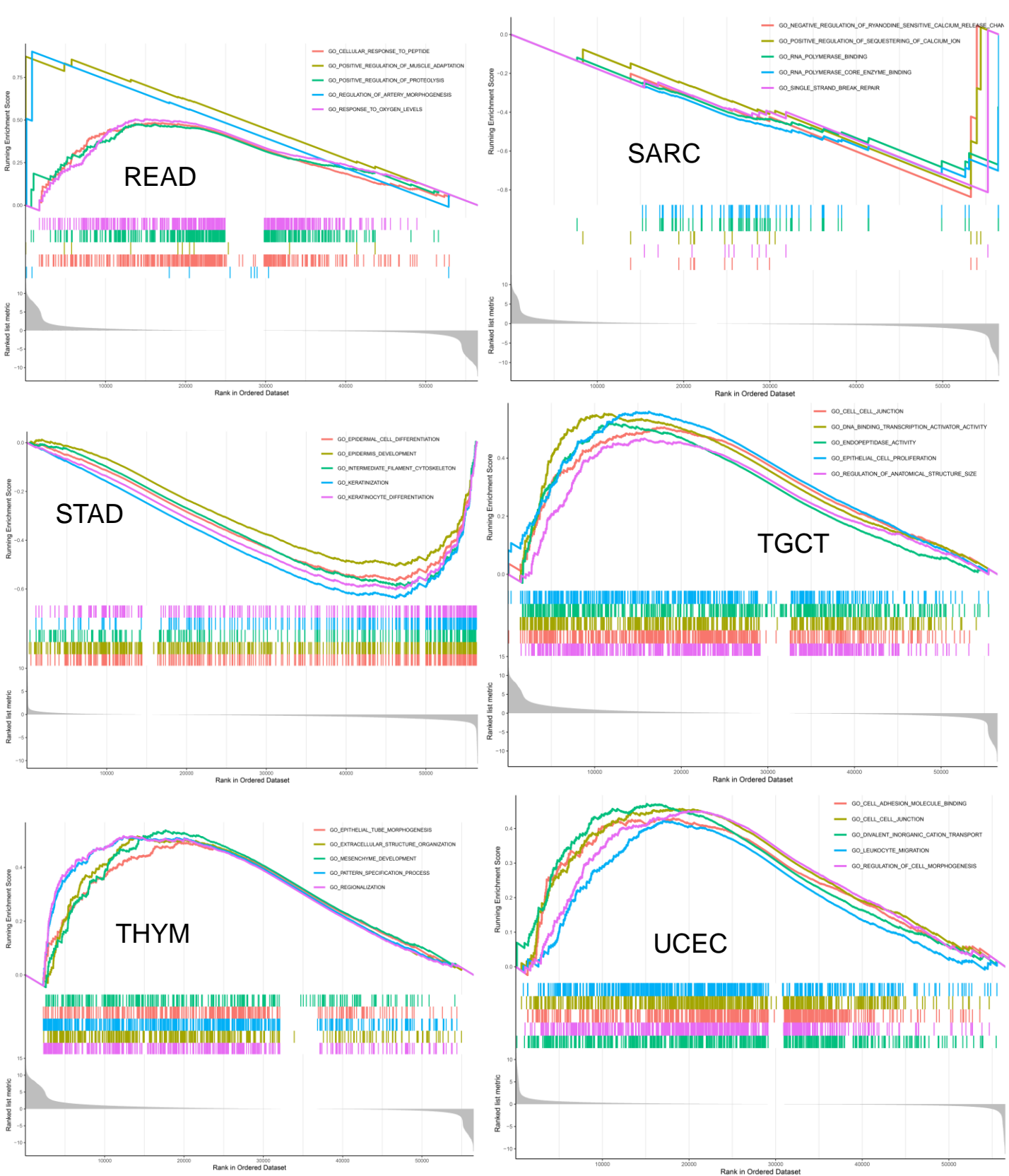

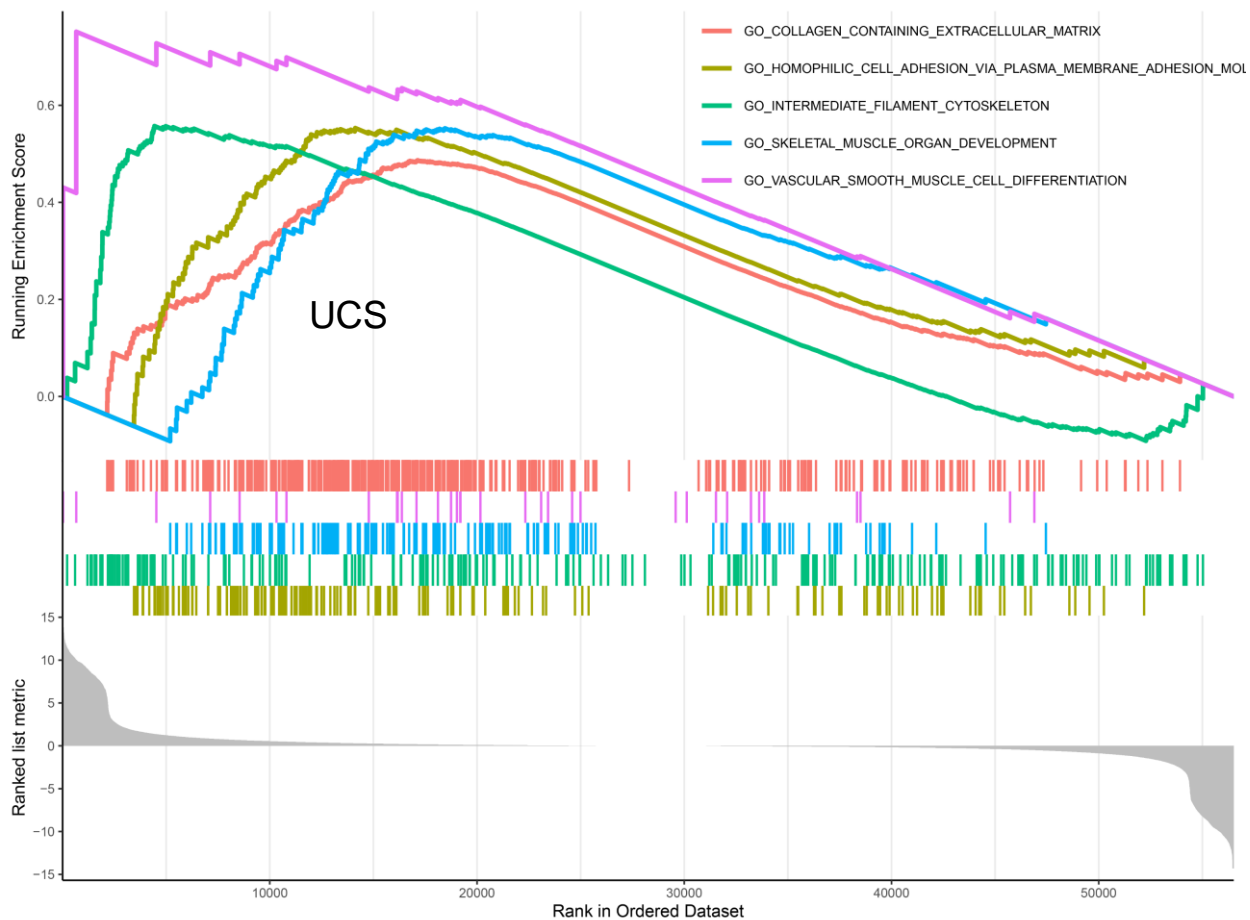

# KEGG

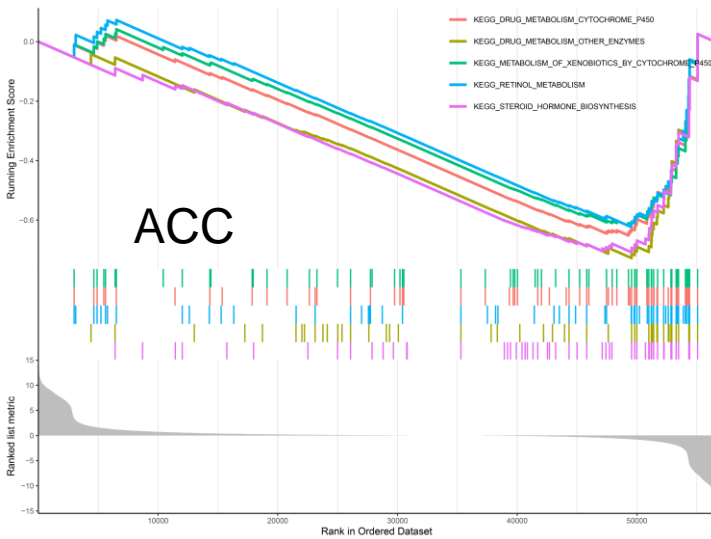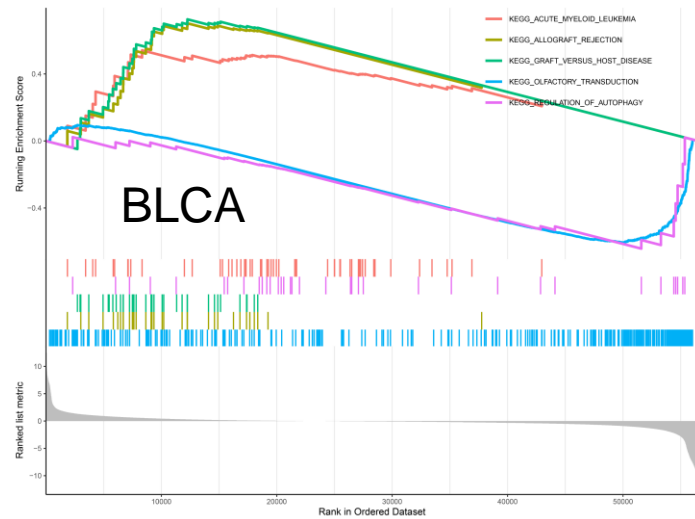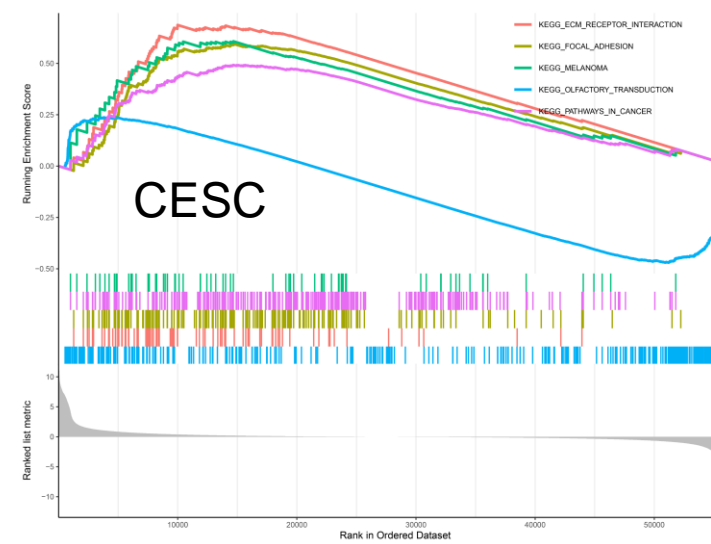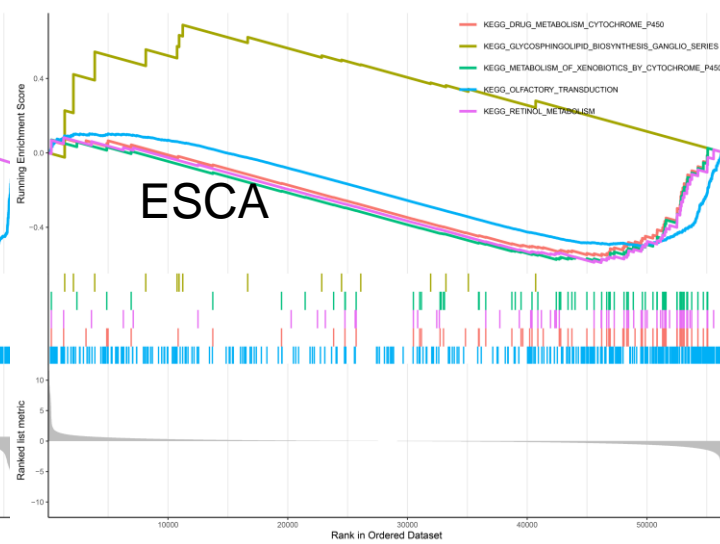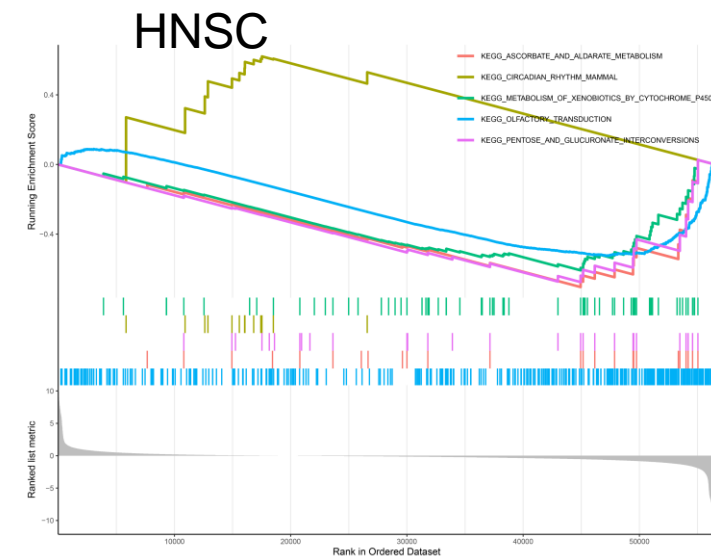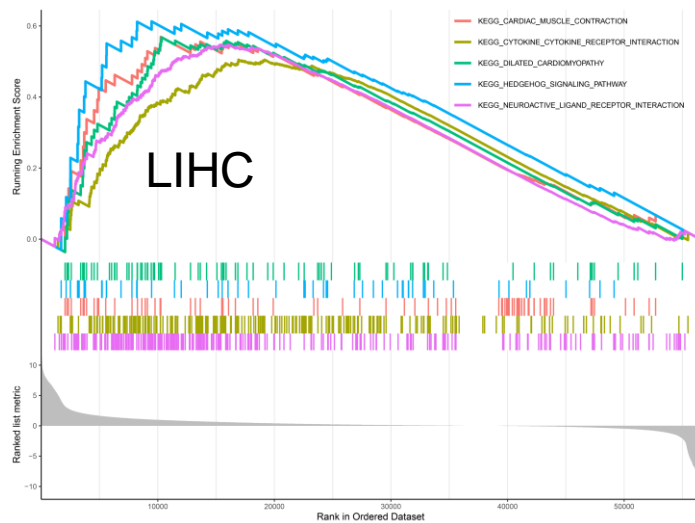

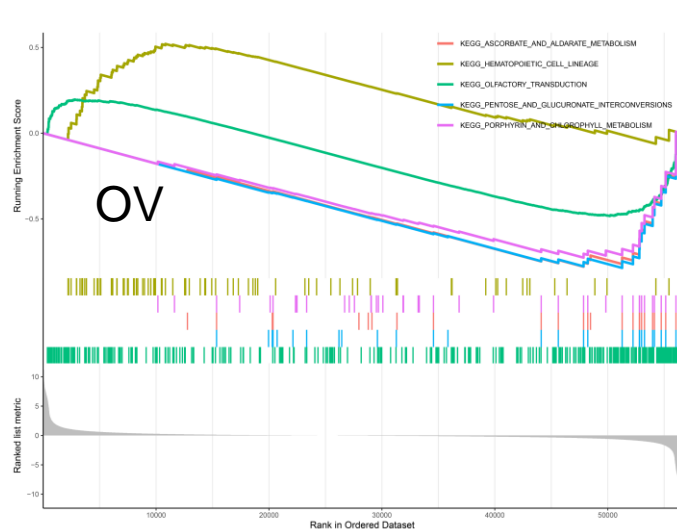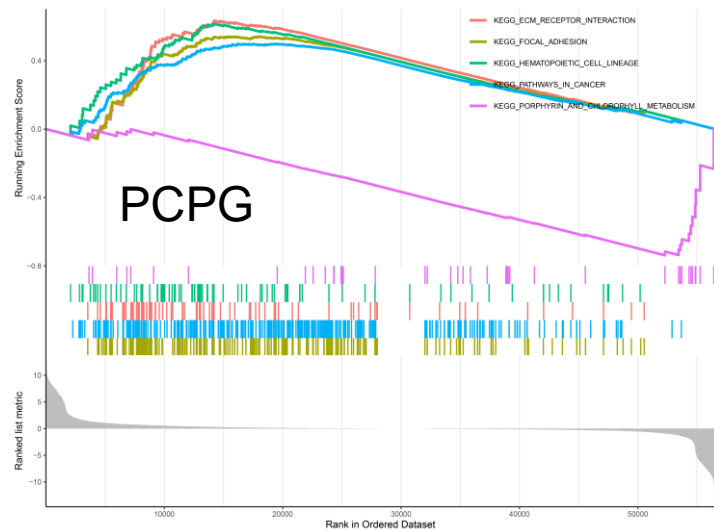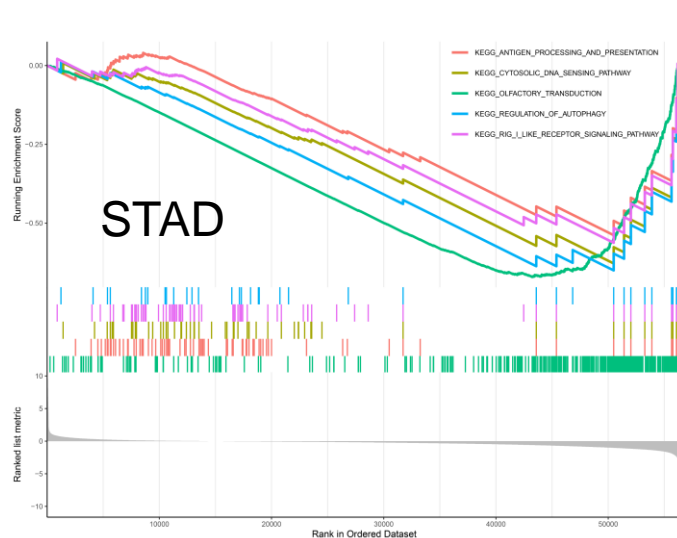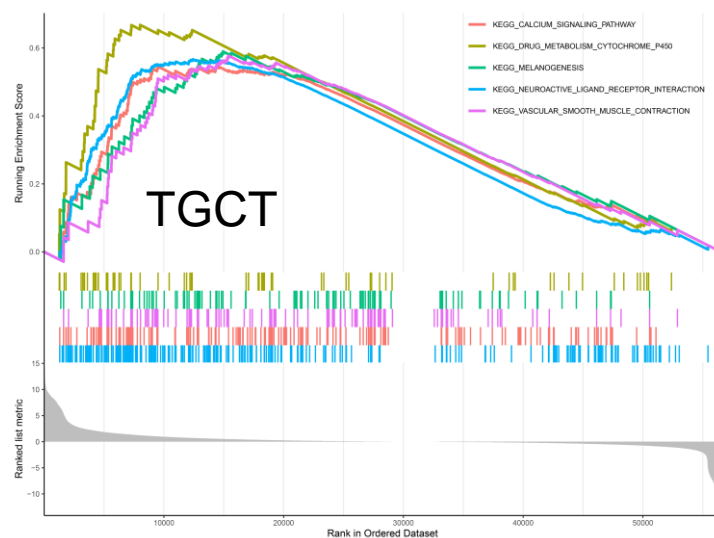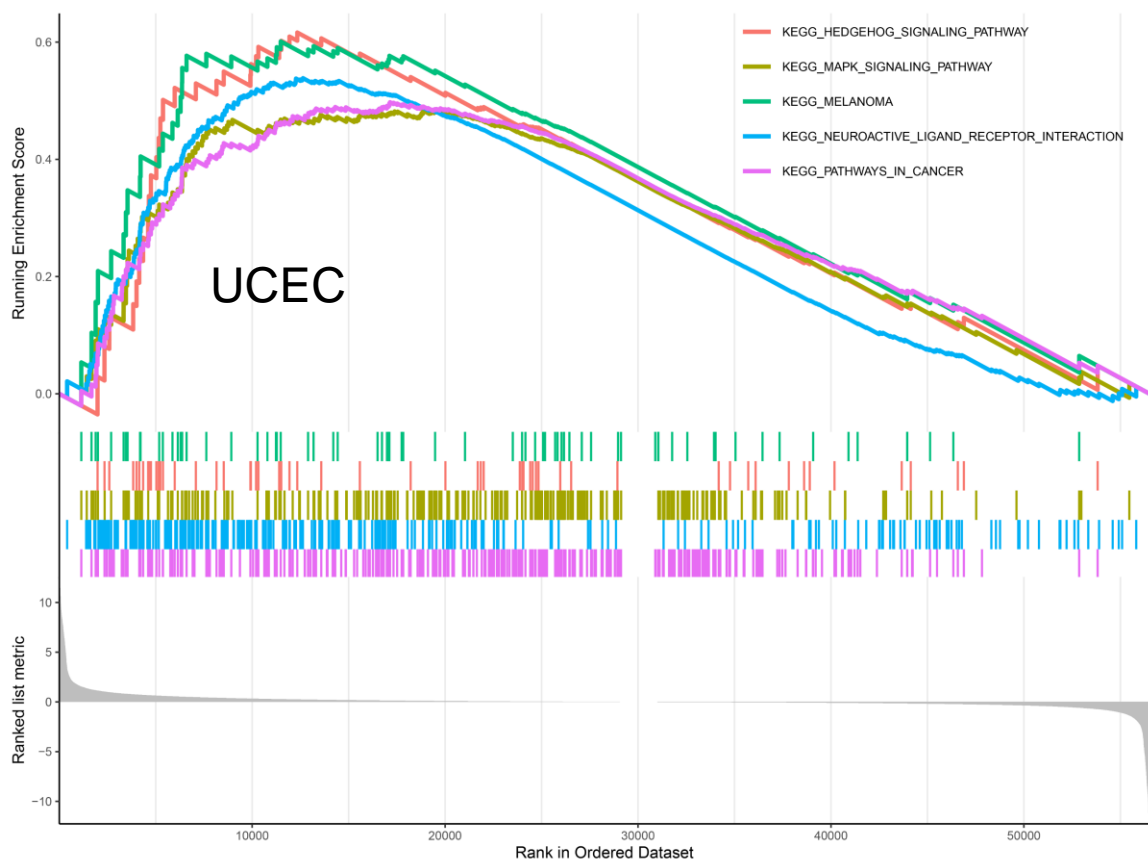

Supplement: Supplementary Materials — Table S1: the infiltration of TIIC correlated with COL5A1 expression in various cancer types. Figure S1: representative clinicopathological photographs of the tumor and paracancerous tissues. Figure S2: correlation between age and COL5A1 expression in patients with various cancer types. Figure S3: association between COL5A1 expression and the tumor stage. Figure S4: correlation between COL5A1 expression and stromal or immune scores. Figure S5: GO and KEGG pathway analyses of COL5A1 in various tumors. [file 6419695.f1.zip › Figure S5 (1).pdf]
